# IL-4 and arginine synergy accelerates early bone regeneration via enhanced efferocytosis and ameliorated microenvironment

**DOI:** 10.1016/j.isci.2026.117739

**Published:** 2026-09-22

**Authors:** Lingxi Meng, Rongpu Liu, Siyi Wang, Junyi Wang, Ilya A. Vinnikov, Rong Yang, Xinyu Song, Hongming Zhang, Jiajia Wang, Wenjie Zhang

**Affiliations:** 1 Department of Prosthodontics, Shanghai Ninth People’s Hospital, Shanghai Jiao Tong University School of Medicine, College of Stomatology, Shanghai Jiao Tong University, National Center for Stomatology, National Clinical Research Center for Oral Diseases, Shanghai Key Laboratory of Stomatology, Shanghai Research Institute of Stomatology, Shanghai, China

**Keywords:** implant, macrophages, efferocytosis, immunomodulation, osteogenic microenvironment

## Abstract

Host bone microenvironment is a critical factor for successful bone regeneration during implantation. In this work, we identified a spatiotemporal mismatch at drilling-induced injury sites where initial apoptosis outpaces macrophage recruitment, leading to apoptotic cell accumulation and oxidative stress that impairs osteogenic differentiation. To mitigate this phenomenon, we developed a synergistic strategy co-delivering interleukin 4 (IL-4) and arginine (Arg) to reprogram the microenvironment. Mechanistically, IL-4 recruits and polarizes macrophages toward a pro-reparative M2 phenotype, while Arg fuels the arginase-1 metabolic pathway to sustain continual efferocytosis and secretion of pro-regenerative cytokines. In a mouse bone injury model, transcriptomic and histological analyses confirmed that this strategy accelerates apoptotic clearance and establishes an M2-dominant environment, significantly enhancing early bone regeneration. Altogether, this work proposes a novel therapeutic paradigm targeting macrophage efferocytosis to resolve early microenvironmental imbalances, providing a promising therapeutic approach to promote bone defect healing and offering novel insights into optimizing host microenvironments for osseointegration following implantation.

## Highlights

• Apoptosis at bone defects drives an anti-osteogenic oxidative stress environment
• IL-4 drives a pro-reparative macrophage phenotype and upregulates the Arg1 pathway
• Exogenous Arg fuels efferocytosis and secretion of pro-regenerative factors
• Co-delivery of IL-4 and Arg via injectable hydrogel accelerates bone regeneration

## Graphical abstract

**Figure:**
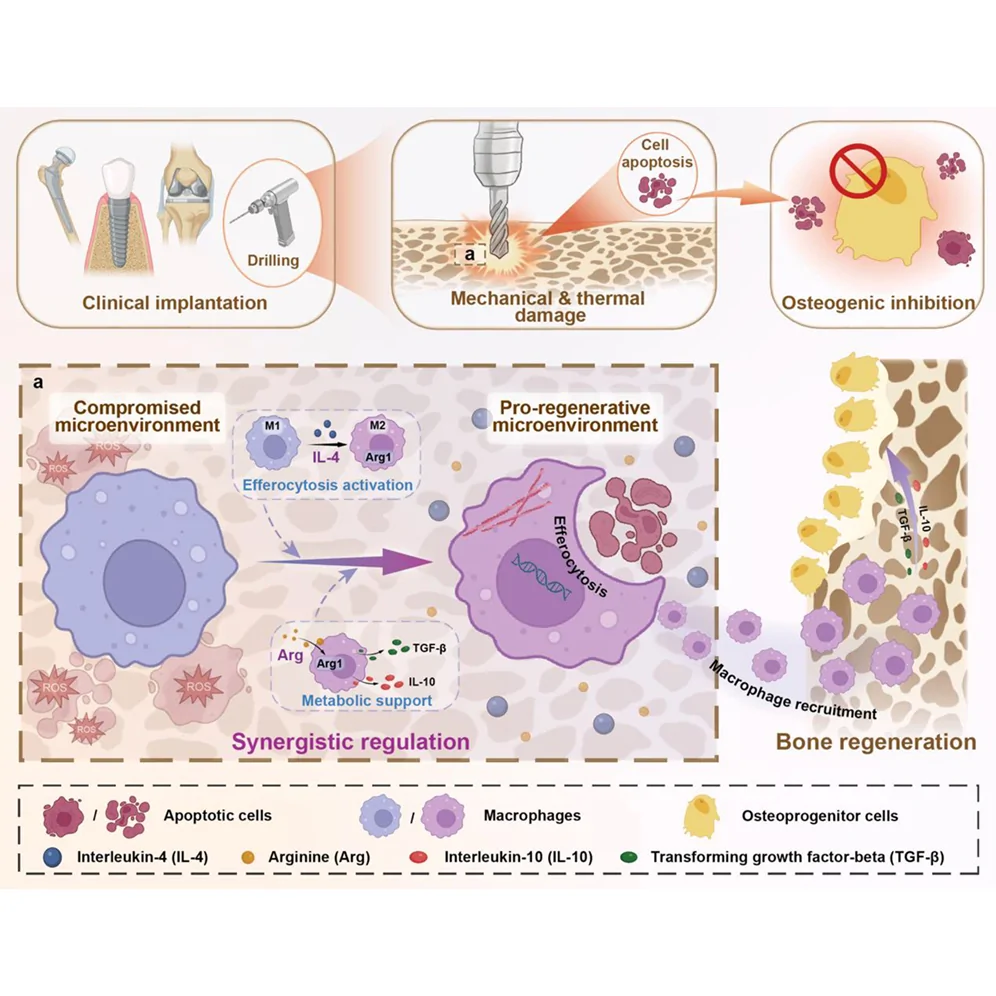

## Teaser

Musculoskeletal medicine; Immune response; Components of the immune system

## Introduction

Implants are widely used in dentistry and orthopedic surgery.^1^ Successful implantation depends on rapid osseointegration, which bridges the physical gap between the implant surface and the host bone bed during the early stages of implantation.^2^ Mechanistically, this integration involves two distinct pathways: (1) contact osteogenesis, which occurs directly on the implant surface, and (2) distance osteogenesis, which originates from the host bone surface.^3^^,^^4^ These two processes proceed bidirectionally—growing outward from the implant and inward from the bone—to fill the interfacial gap and jointly establish stable bone regeneration.^5^ Extensive research currently focuses on surface modifications to optimize contact osteogenesis by enhancing cell adhesion and bone formation directly on the implant.^6^^,^^7^^,^^8^ While cytokine-loaded coatings represent a significant advancement, they function primarily as passive scaffolds awaiting cell colonization. Crucially, these surface-centric strategies overlook the pathological state of the surrounding host bone and fail to actively recondition the compromised microenvironment—specifically, clearing the apoptosis-rich zone created by drilling.^9^ Indeed, during implant site preparation, drilling inevitably causes friction and mechanical damage to the bone tissue.^10^ This in turn leads to the death of osteogenic cells in the surrounding bone, creating a zone of apoptotic injury that causes macrophages to divert significant energy and resources toward clearing these apoptotic cells (ACs).^11^^,^^12^^,^^13^ Consequently, the resulting inflammatory microenvironment leads to increased apoptosis and suppresses the expression of key osteogenic genes,^14^^,^^15^ thereby delaying the initiation of bone repair. Without addressing this pre-existing burden, surface modifications alone are insufficient to promote distance osteogenesis.

Therefore, accelerating the clearance of the apoptotic burden is not merely about inflammation resolution, but rather a prerequisite for unlocking the regenerative potential of distance osteogenesis. In normal conditions, the clearance of ACs mainly relies on efferocytosis.^16^^,^^17^ During this process, macrophages selectively phagocytize ACs and undergo M2 polarization,^18^ which in turn activates anti-inflammatory signaling pathways and enhances their efferocytic capacity.^19^^,^^20^ This positive feedback loop may promote the transition from the acute inflammatory phase to the tissue repair phase. However, during implant placement, immediate damage leads to the early appearance of a large number of ACs,^21^ while macrophage recruitment and polarization often lag behind this rapid burst of apoptosis.^22^ This delay results in secondary necrosis and persistent inflammation within the early inflammatory microenvironment.^23^ Concurrently, exposure to the early inflammatory microenvironment leads to the accumulation of intracellular oxidative stress, which obstructs the function of bone marrow mesenchymal stem cells (BMSCs) and inhibits the continual phagocytic function of macrophages.^24^^,^^25^^,^^26^ Therefore, maintaining sustained and efficient macrophage efferocytosis is crucial for reversing the inflammatory microenvironment and initiating early bone regeneration. While recent studies have utilized cytokine-loaded implant coatings to promote macrophage polarization and improve the osteogenic microenvironment,^21^^,^^27^ these approaches have not addressed the impact of ACs on bone surface osteogenesis, nor have they promoted continuous efferocytosis in the damaged microenvironment to drive the positive feedback effect.^28^

To address this challenge, we designed a synergistic strategy utilizing interleukin 4 (IL-4) and arginine (Arg) to remodel macrophages and reverse the microenvironment, thereby promoting early bone regeneration at the drilling injury site. As a cytokine, IL-4 not only effectively induces macrophage polarization toward the anti-inflammatory M2 phenotype but also possesses significant chemotactic activity.^29^ Arg, as a semi-essential amino acid, is not merely a substrate for arginase-1 (Arg1) but also a rate-limiting metabolic fuel indispensable for sustained efferocytosis.^30^^,^^31^ Recent studies have elucidated a critical metabolic-signaling link in efferocytosis: Arg is converted by Arg1 into polyamines, which subsequently activate Rac1 to drive actin polymerization.^32^^,^^33^ By adding exogenous Arg to fuel the ornithine-polyamine axis, we aim to metabolically reprogram macrophages to sustain their clearance capacity even in the nutrient-deprived injury microenvironment. Critically, we employ IL-4 as a signaling molecule to recruit macrophages and guide them toward reparative polarization, while exogenous Arg serves as a critical “metabolic fuel” to support their proliferation and provide energy for continual efferocytosis. In subsequent *in vivo* studies, by delivering these two agents directly to the bone injury site, we aim to accelerate the clearance of ACs, thereby eliminating osteogenic inhibitory factors and creating a favorable regenerative microenvironment for the migration and osteogenic differentiation of BMSCs.

In this study, we targeted a spatiotemporal mismatch between macrophage recruitment and apoptosis, which inhibits osteogenesis. We demonstrated that the combination of IL-4 and Arg significantly improves macrophage migration, proliferation, and phagocytic capacity *in vitro*, while promoting the secretion of osteogenesis-related cytokines. Finally, using a mouse femoral defect model, we confirmed *in vivo* that the simultaneous delivery of IL-4 and Arg effectively promotes *in situ* M2 macrophage dominance and significantly enhances new bone formation. Altogether, this study proposes a novel therapeutic paradigm for immune cell-regulated osteogenesis, in which the enhancement of efferocytosis optimizes the early immune-metabolic microenvironment, thereby significantly accelerating distance osteogenesis.

## Results

### Spatiotemporal mismatch within the bone injury site

To evaluate the dynamic interplay between local apoptosis and macrophage recruitment following bone injury, we established a cortical bone defect model (Figure 1A). Interestingly, in the absence of water cooling, apoptotic signals accumulated not only on the damaged surface of the implant site but also into the deep regions of the bone tissue, highlighting the severe destructive effect of thermal injury. Conversely, while water cooling markedly reduced the depth of apoptosis, a substantial number of ACs remained concentrated at the superficial layer, indicating that mechanical stress-induced injury inevitably damages peripheral bone cells (Figure 1B).

**Figure 1:**
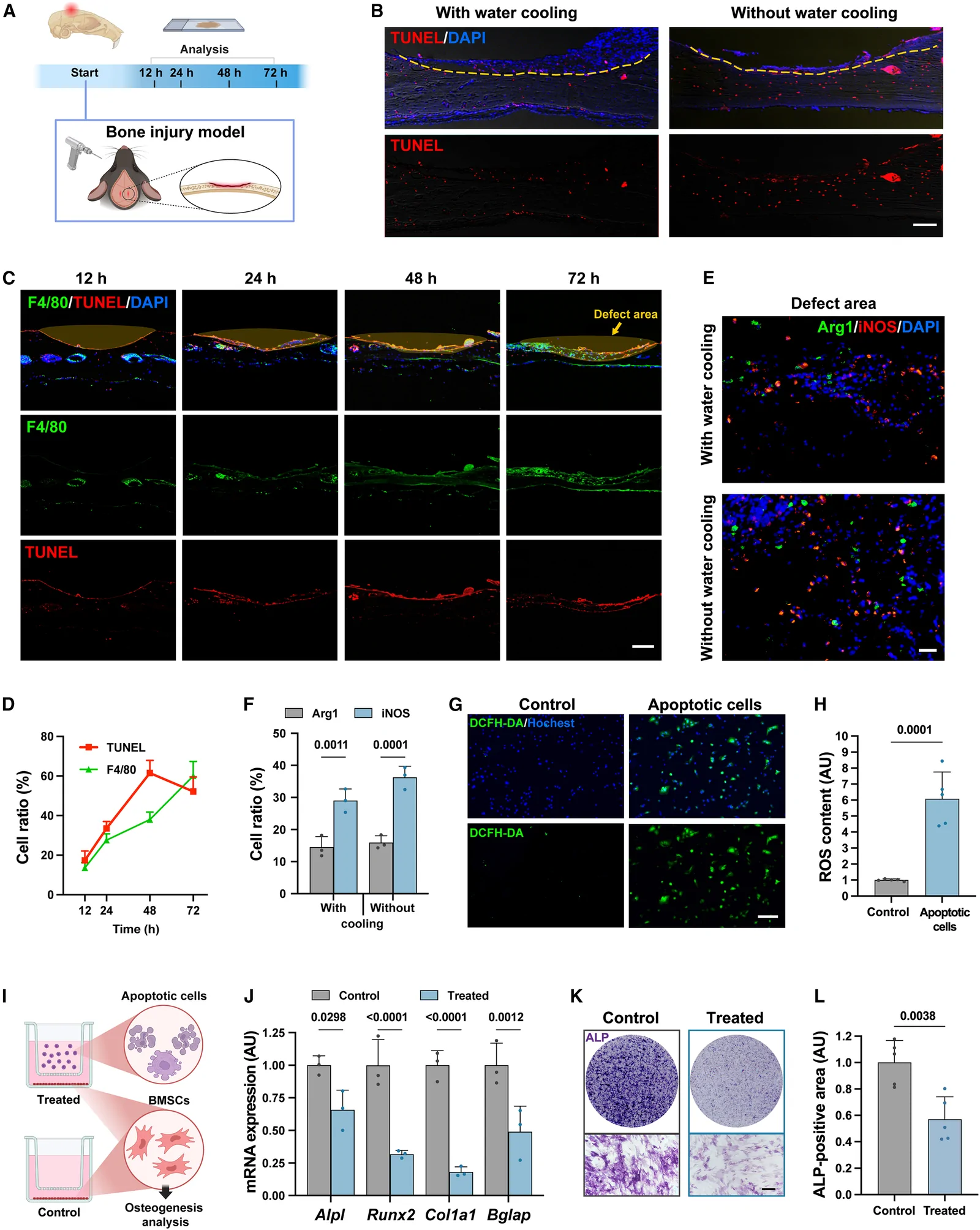
Injury-evoked apoptosis impedes osteogenesis (A) Schematic illustration of the bone injury model. (B) Representative images of the bone injury site with or without water cooling during the drilling process. (C and D) Spatiotemporal distribution of macrophages and apoptotic cells at the bone injury site, showing representative fluorescence images of macrophages (F4/80) and apoptotic cells (terminal deoxynucleotidyl transferase dUTP nick-end labeling [TUNEL]) at 12, 24, 48, and 72 h post-surgery (C) and quantification of the percentages of TUNEL^+^ and F4/80^+^ cells over time (D). (E and F) Phenotypic characterization of early-recruited macrophages at 72 h post-surgery, showing representative immunofluorescence images of Arg1 and iNOS expression with or without water cooling (E) and quantification of the percentages of Arg1^+^ and iNOS^+^ cells in the cooling and without-cooling groups (F). (G and H) Reactive oxygen species (ROS) detection and quantification in apoptotic cells, showing representative fluorescence images of ROS accumulation (G) and quantification of ROS fluorescence intensity (H). (I) Schematic illustration of the *in vitro* co-culture system for investigating the influence of apoptotic cells on osteogenic differentiation: bone marrow mesenchymal stem cells (BMSCs) were treated with apoptotic cells (treated) or a normal medium (control). (J) Quantitative reverse-transcription PCR (RT-qPCR) analysis of osteogenic-related gene expression in BMSCs following 3 days of co-culture. (K and L) Evaluation of early osteogenic differentiation, showing representative alkaline phosphatase (ALP) staining images (K) and quantification of the relative ALP-positive area in BMSCs after 7 days of induction (L). Error bars represent standard deviation (SD). Data were analyzed by two-way ANOVA followed by Sidak’s multiple comparisons test (D) or unpaired two-tailed Student’s *t* test (F, H, J, and L). Sample sizes: *n* = 3 animals per group (B–F); *n* = 5 biological replicates (G and H); *n* = 3 (J) and 5 (K and L) biological replicates, each performed with 3 technical replicates. Scale bars, 100 μm (B and K), 500 μm (C), 50 μm (E), and 200 μm (G).

Subsequent time-course analysis revealed a distinct spatiotemporal mismatch between AC accumulation and macrophage recruitment. Specifically, while ACs emerged as early as 12 h post-surgery, reaching a plateau at 48 h, the incremental recruitment of F4/80^+^ macrophages was delayed (Figures 1C and 1D). Consequently, this spatiotemporal mismatch leads to the accumulation of uncleared ACs, thereby potentially aggravating the inflammatory microenvironment and delaying the initiation of effective bone repair.^23^

Interestingly, 72 h after surgery, the pro-inflammatory M1 phenotype prevailed over the M2 among the recruited macrophages, based on the labeling by iNOS and Arg1, respectively (Figures 1E and 1F). This suggests that the early accumulation of ACs drives the formation of a hostile pro-inflammatory microenvironment.

To systematically evaluate the impact of this apoptotic burden on subsequent osteogenesis, we established an *in vitro* model to mimic the specific cellular damage induced by implant drilling (Figure S1). Fluorescence analysis showed a marked increase in reactive oxygen species (ROS) within the ACs compared to the control, confirming the accumulation of severe oxidative stress (Figures 1G and 1H).

To assess how this microenvironment affects bone regeneration, we co-cultured mouse BMSCs with these ACs (Figure 1I). Consistent with previously reported effects of the elevated ROS levels,^24^^,^^25^^,^^26^ exposure to ACs significantly suppressed the mRNA expression of key osteogenic markers *Alpl*, *Runx2*, *Col1a1*, and *Bglap* (Figure 1J) and the alkaline phosphatase (ALP) activity of BMSCs (Figures 1K and 1L). Altogether, these findings demonstrate that the bone injury area is characterized by a microenvironment involving AC accumulation, induction of M1 pro-inflammatory macrophage polarization, and oxidative stress, leading to the impairment of osteoprogenitor cell differentiation.

### IL-4 and Arg synergistically remodel macrophages

To delineate the regulatory impact of IL-4 on macrophage function, we first sought to assess transcriptomic changes within the injury site. Notably, IL-4 potently elevated classical M2-type markers *Chil3*, *Arg1*, and *Retnla* as well as key genes involved in cellular clearance and metabolism, such as *Msr1*, *Marco*, *Sod2*, and *Aif1*, while down-regulating the M1-related genes *Prok2*, *Mamdc2*, *Btg2*, *and Has2* (Figures 2A–2C). Cytokine-cytokine receptor interaction, JAK-STAT signaling, Arg and proline metabolism, phagosome, and peroxisome were significantly enriched by IL-4 (Figure 2D), in agreement with previous reports of this cytokine.^34^^,^^35^^,^^36^ The enrichment of the latter two pathways highlights the key role of IL-4 in enhancing intracellular degradation of ACs and ROS clearance. Furthermore, the up-regulation of the Arg metabolism pathway motivated us to investigate the synergistic effects between IL-4 and Arg, paving the way to the strategy of their simultaneous application to remodel the macrophages (Figure 2E).

**Figure 2:**
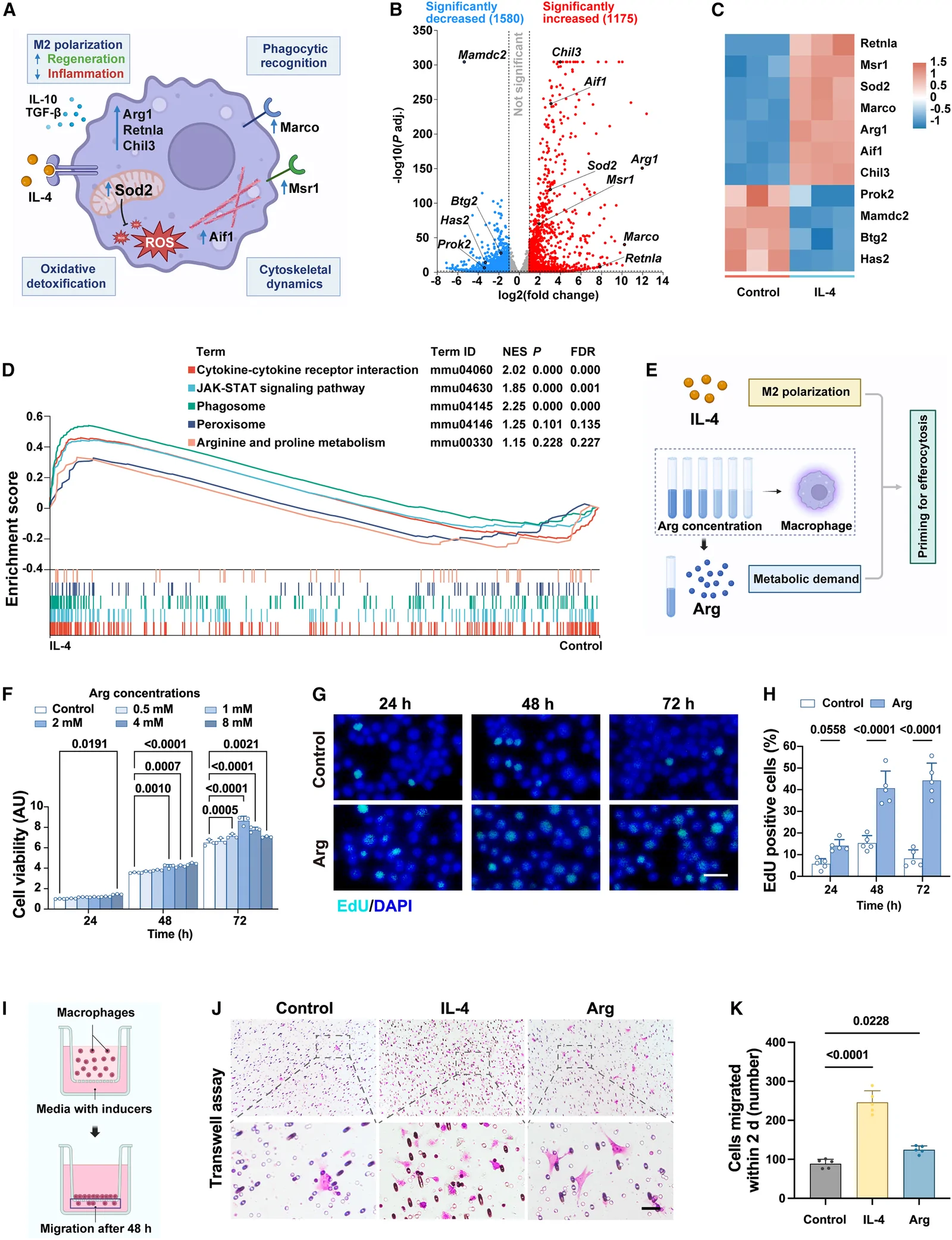
IL-4 and Arg remodel macrophages and promote their migration (A) Schematic illustration of the regulatory role of IL-4 on macrophage phenotype. (B) Volcano plot displaying differentially expressed genes (DEGs) between the control and IL-4-treated macrophages. (C) Heatmap demonstrating the expression of key macrophage polarization markers. (D) Multi-pathway Gene Set Enrichment Analysis (GSEA) demonstrating the integrated enrichment of indicated pathways regulating macrophage functions. (E) Schematic illustration of macrophage priming for efferocytosis mediated by IL-4 and Arg. (F) Relative cell viability of macrophages treated with different concentrations of Arg at 24, 48, and 72 h. (G and H) Macrophage proliferation evaluated by 5-ethynyl-2′-deoxyuridine (EdU) assay, showing representative fluorescence images of EdU and 4',6-diamidino-2-phenylindole (DAPI) staining at 24, 48, and 72 h (G) and quantification of EdU-positive cells treated with 2 mM Arg (H). (I) Schematic illustration of the Transwell migration assay. (J and K) Macrophage recruitment evaluated by Transwell assay, showing representative crystal violet staining images of migrated macrophages after 48 h (J) and quantification of the number of migrated macrophages (K). Error bars represent SD. Data were analyzed by two-way ANOVA followed by Sidak’s multiple comparisons test (F and H) or one-way ANOVA followed by Tukey’s post hoc test (K). Sample sizes: *n* = 3 biological replicates (B–D and F); *n* = 5 biological replicates, each performed with 3 technical replicates (G, H, J, and K). Scale bars, 25 μm (G), 500 μm (J, top), and 100 μm (J, bottom).

Next, we identified 2 mM Arg as the optimal pro-survival concentration for macrophages (Figure 2F), confirmed by 5-ethynyl-2′-deoxyuridine (EdU) labeling to effectively promote their proliferation (Figures 2G and 2H). Interestingly, both Arg and especially IL-4 significantly increased the recruitment and migration of macrophages (Figures 2I–2K). In summary, these results indicate that IL-4 promotes a pro-regenerative environment by enhancing phagocytosis and oxidative detoxification, while the optimal supplementation of Arg enhances viability and promotes macrophage recruitment, providing a mechanistic basis for the subsequent synergy in macrophage remodeling.

### Simultaneous application of IL-4 and Arg stimulates efferocytosis and regulates microenvironment

Next, we functionally assessed the individual and synergistic effects of both compounds on the clearance of ACs (Figure 3A).^37^^,^^38^^,^^39^ Interestingly, while IL-4 alone, but not Arg, enhanced efferocytosis by 1.7-fold, their simultaneous application further increased the AC clearance, more than doubling it compared with the untreated macrophages (Figures 3B and 3C), consistent with the cell tracking analysis (Figure 3D). Further experiments demonstrated that the IL-4 + Arg combination significantly increased the phagocytic efficiency as early as 6 h (Figure 3E; Figure S2). This elevated phagocytic capacity peaked at 24 h and was sustained through 48 h, whereas that of the control group exhibited a marked decline at 48 h. Altogether, these results indicate that the simultaneous IL-4 + Arg treatment is essential for timely apoptotic debris removal and subsequent resolution of inflammation in the injured microenvironment. Next, we investigated the subsequent microenvironmental remodeling associated with efferocytosis and the secretion of anti-inflammatory cytokines (Figure 3F). In agreement with our previous results, simultaneous application of IL-4 and Arg, but not each of them individually, reduced ROS levels to approximately 30% of those in the control group and increased the secretion of TGF-β1 and IL-10, the key anti-inflammatory and pro-regenerative cytokines (Figures 3G and 3H). Altogether, these findings indicate that the synergistic IL-4 and Arg strategy effectively enhances macrophage efferocytosis, promotes the secretion of pro-regenerative factors, and suppresses microenvironmental oxidative stress, thereby establishing a favorable microenvironment for subsequent tissue repair.

**Figure 3:**
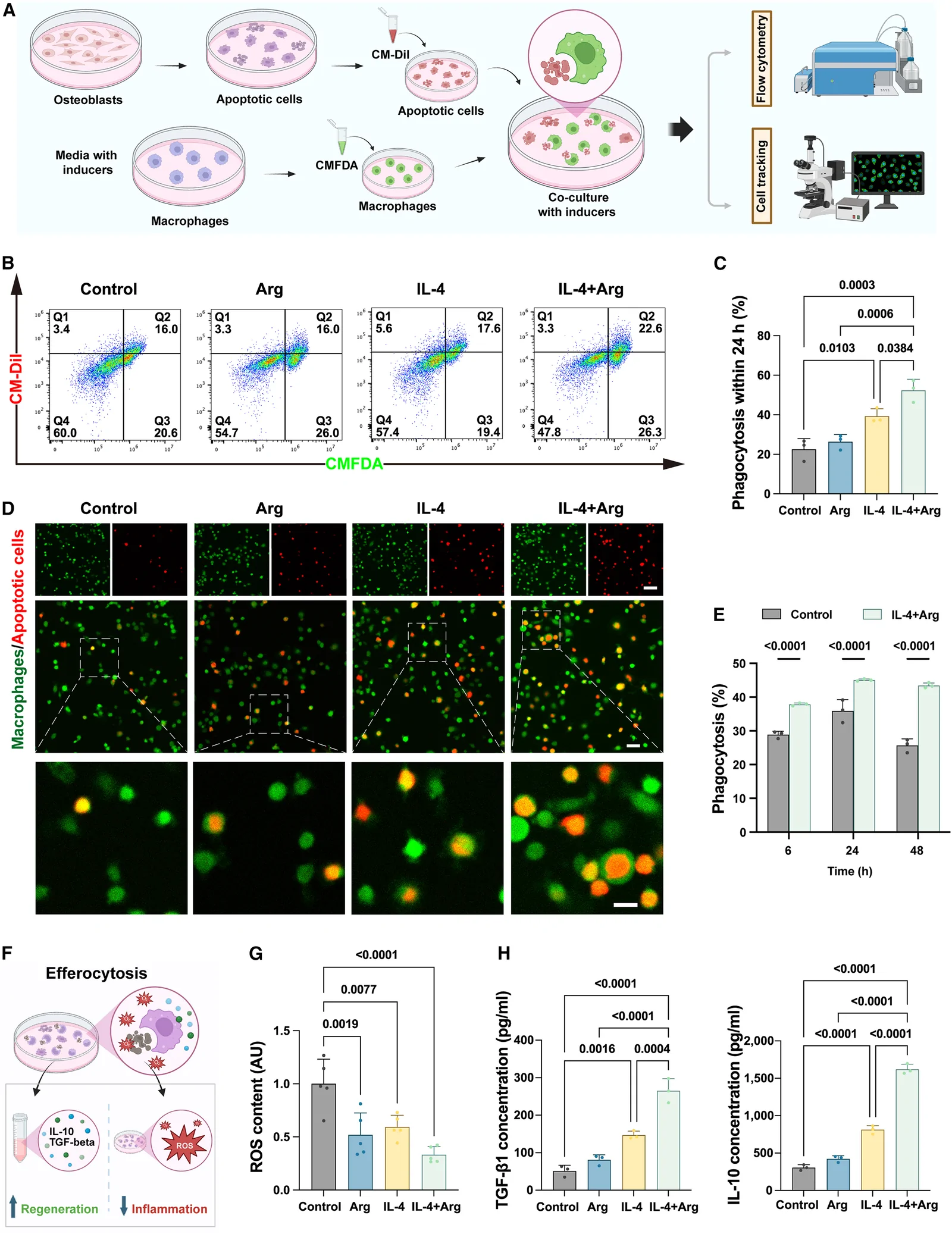
Simultaneous application of IL-4 and Arg promotes efferocytosis and improves the pro-regenerative microenvironment (A) Schematic illustration of the *in vitro* efferocytosis assessment. (B and C) Macrophage efferocytosis assessment via flow cytometry, showing representative scatterplots of macrophages engulfing apoptotic cells (B) and quantification of the efferocytosis rate after 24 h (C). (D) Morphological evaluation of macrophage efferocytosis, showing representative cell tracking images of macrophages (labeled with CMFDA) engulfing apoptotic cells (labeled with CM-Dil) after 24 h of co-culture. (E) Quantification of the efferocytosis rate after 6, 24, and 48 h determined by flow cytometry under Control and IL-4 + Arg conditions. (F) Experimental design for detecting microenvironmental changes following efferocytosis. (G) ROS quantification following efferocytosis. (H) Enzyme-linked immunosorbent assay (ELISA) quantification of TGF-β1 and IL-10 in the culture supernatants. Error bars represent SD. Data were analyzed by one-way ANOVA followed by Tukey’s post hoc test (C, G, and H) or two-way ANOVA followed by Sidak’s multiple comparisons test (E). Sample sizes: *n* = 3 biological replicates (B–E); *n* = 5 biological replicates, each performed with 3 technical replicates (G) and *n* = 3 biological replicates, each performed with 3 technical replicates (H). Scale bars, 100 μm (D) and 20 μm (inset).

To elucidate the molecular mechanisms underlying these synergistic effects of IL-4 and Arg on macrophages, we first evaluated the expression of pro-regenerative cytokines and macrophage polarization markers.^40^ In agreement with our data, *Tgfb1* and *Il10* levels in the macrophages were effectively upregulated by the simultaneous IL-4 + Arg treatment. Moreover, while IL-4 alone, but not Arg, upregulated the classical M2 markers *Arg1* and *Cd206*, their combination further promoted macrophage remodeling (Figure 4A). These data further confirm that such combined stimulation synergistically predisposes a transition toward a functionally mature, reparative M2 state of the macrophages.

**Figure 4:**
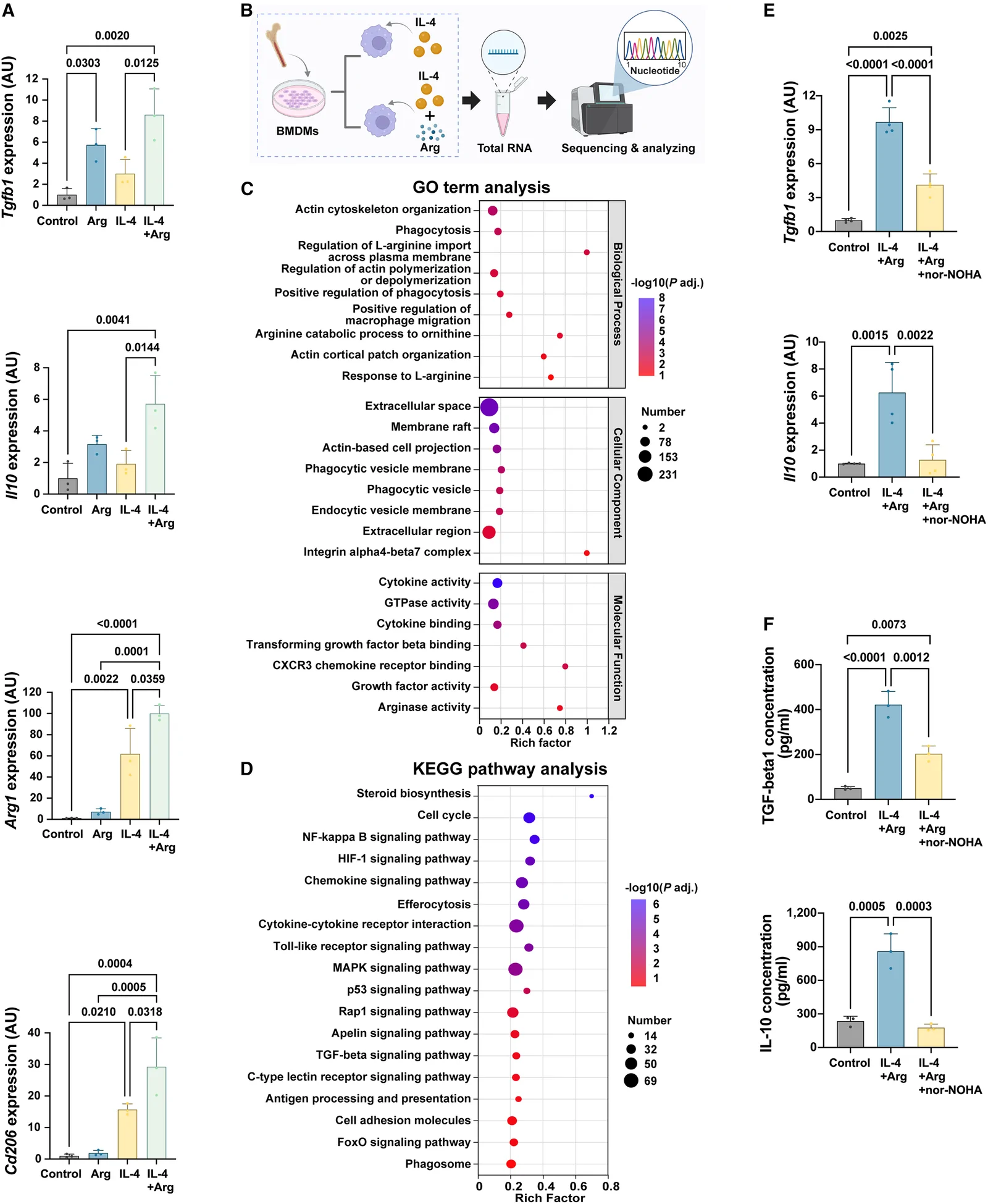
Transcriptomic changes underlying synergistic regulation of macrophages by IL-4 and Arg (A) RT-qPCR analysis of M2-related marker gene expression (*Tgfb1*, *Il10*, *Arg1*, and *Cd206*) in macrophages. (B) Schematic illustration of the bone marrow-derived macrophages (BMDMs) isolation, total RNA extraction, and transcriptomic sequencing workflow. (C) Functional annotation of upregulated genes using Gene Ontology (GO) enrichment analysis. (D) Kyoto Encyclopedia of Genes and Genomes (KEGG) pathway enrichment analysis emphasizing significant signaling pathways. (E) RT-qPCR analysis of *Tgfb1* and *Il10* mRNA expression levels of macrophage after treatment with the IL-4 + Arg combination in the presence or absence of nor-NOHA. (F) ELISA quantification of secreted TGF-β1 and IL-10 protein concentrations in the culture supernatants under nor-NOHA inhibition. Error bars represent SD. Data were analyzed by one-way ANOVA followed by Tukey’s post hoc test (A, E, and F). Sample sizes: *n* = 3 biological replicates (C and D); *n* = 3 (A and F) and 4 (E) biological replicates, each performed with 3 technical replicates.

To systematically assess the additive effect of Arg supplementation compared to IL-4 alone on bone marrow-derived macrophages (BMDMs), we performed transcriptome profiling followed by Gene Ontology (GO) enrichment analysis for genes upregulated by the IL-4 + Arg treatment (Figures 4B and 4C; Figure S3). Interestingly, this synergistic treatment enriched genes associated with actin cytoskeleton, efferocytosis, and Arg metabolism among the most differentially regulated biological processes; extracellular space, cell surface, membrane rafts, and vesicles among cellular components; cytokines and growth factors among molecular functions. The latter two categories feature terms critical for macrophage recognition and phagocytosis, as well as secretory activity,^41^^,^^42^ which might be associated with the increased release of pro-reparative factors observed in our earlier experiments. These results imply that in combination with IL-4, Arg empowers macrophages with superior cytoskeletal reorganization for promoting phagocytosis and secretion, thus facilitating more efficient AC clearance.

In agreement with the accelerated AC clearance and cytokine secretion profile observed experimentally, Kyoto Encyclopedia of Genes and Genomes (KEGG) pathway analysis of genes upregulated by the IL-4 + Arg treatment revealed efferocytosis, phagosome, JAK-STAT, chemokine, and TGF-β signaling among the top pathways underlying this synergistic effect (Figure 4D). Altogether, enrichment in genes involved in phagocytic recognition, actin rearrangement, and pro-repair signaling suggests that when subjected to Arg in addition to IL-4, phagocytizing macrophages efficiently secrete potent paracrine signals to induce osteogenic differentiation of BMSCs, critical for bone regeneration.

To validate the critical role of the Arg1 metabolic pathway in the ameliorating effects of the simultaneous IL-4 + Arg treatment, we conducted a pharmacological inhibition experiment utilizing a pan-arginase inhibitor *N*ω-hydroxy-nor-L-arginine (nor-NOHA).^43^ Rewardingly, this inhibitor effectively counter-regulated IL-4 + Arg-mediated transcriptional and secretory upregulation of both TGF-β1 and IL-10 (Figures 4E and 4F), validating the causative function of Arg1 for the pro-regenerative cytokine secretion by macrophages treated with IL-4 + Arg and proving the critical role of the latter in ameliorating cellular metabolism.

### Efferocytosis-mediated pro-osteogenic microenvironment and *in vivo* osteogenic priming

To evaluate the pro-osteogenic effects of enhanced efferocytosis, we subjected osteoprogenitor cells to conditioned media from phagocytizing or control macrophages simultaneously incubated with or without IL-4 and Arg (Figure 5A). In agreement with our previous data, conditioned medium from macrophages treated with IL-4 and Arg significantly promoted the migration of MC3T3-E1 cells and BMSCs (Figures 5B and 5C), with even further potentiation by efferocytosis in the latter cell line, which was mirrored by the levels of ALP activity and osteogenesis-related genes *Alpl*, *Runx2*, *Col1a1*, and *Bglap* (Figures 5D–5I). These results further confirm that the IL-4 + Arg treatment strategy effectively converts an apoptosis-poisoned microenvironment into a regenerative one, fostering both migration and differentiation of osteogenic cells.

**Figure 5:**
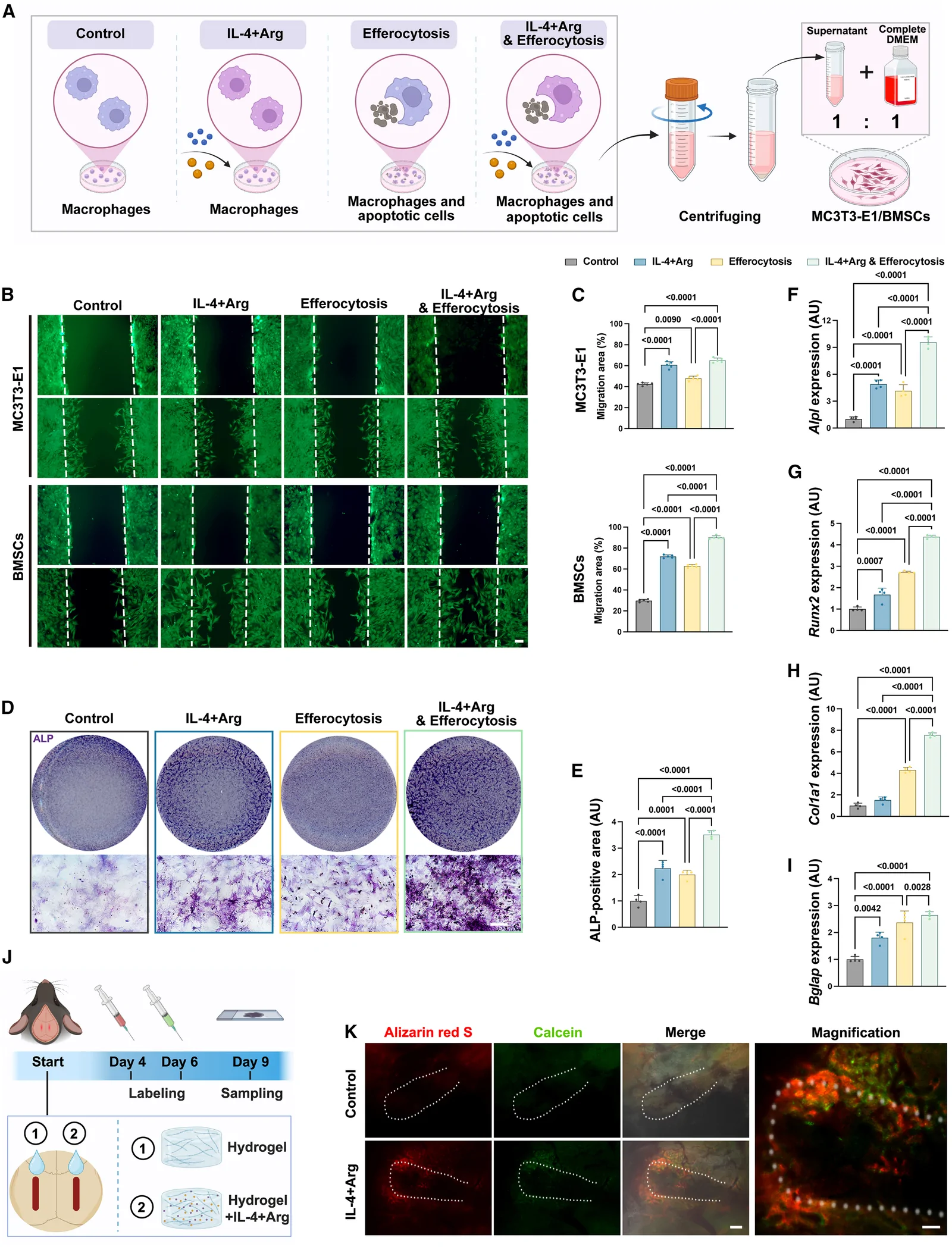
Efferocytosis-remodeled microenvironment promotes osteogenic priming (A) Schematic illustration of the preparation of conditioned media from macrophages following the efferocytosis process. (B and C) Cell migration assessment via scratch wound healing assay in MC3T3-E1 and BMSCs populations, showing representative images (B) and quantification of the migration area after 24 h of treatment (C). (D and E) Early osteogenic differentiation assessment, showing representative images of ALP staining after 7 days of induction (D) and quantification of the relative ALP-positive area (E). (F–I) RT-qPCR analysis of osteogenic-related gene expression (*Alpl*, *Runx2*, *Col1a1*, and *Bglap*) in BMSCs after 3 days of treatment. (J) Schematic illustration of the *in vivo* sequential fluorescent labeling protocol. (K) Representative fluorescence images of the sequential labeling in the bone injury site. Error bars represent SD. Data were analyzed by one-way ANOVA followed by Tukey’s post hoc test (C, E, and F–I). Sample sizes: *n* = 5 biological replicates (C); *n* = 4 biological replicates (E); *n* = 4 biological replicates, each performed with 3 technical replicates (F–I) and *n* = 4 animals per group (K). Scale bars, 100 μm (B and D), 500 μm (K), and 200 μm (inset).

To validate this pro-regenerative transition *in vivo*, we evaluated early bone growth via postoperative sequential fluorescent labeling (Figure 5J). Indeed, simultaneous treatment with IL-4 and Arg significantly enhanced new bone formation both after days 4 and 6, as measured by inclusion of alizarin red S and calcein, respectively (Figure 5K), confirming the efficacy of our synergistic strategy in promoting early osteogenic priming and matrix mineralization *in vivo*.

### Simultaneous application of IL-4 and Arg promotes bone regeneration *in vivo*

To evaluate the *in vivo* therapeutic efficacy of our strategy, we conformally covered the mouse femoral bone defect surface by previously characterized injectable hydrogel system for localized and sustained delivery of IL-4 and Arg, followed by rapid fixation via photo-crosslinking (Figure 6A; Figures S4–S8). In agreement with our *in vitro* data, IL-4 + Arg application effectively and continuously promoted infiltration of the injury site by macrophages as early as day 1 after surgery (Figures 6B and 6C). Notably, in contrast to control defects, many of these macrophages co-expressed Arg1, indicating an M2-dominant environment to promote and maintain the reparative macrophage phenotype *in situ*.

**Figure 6:**
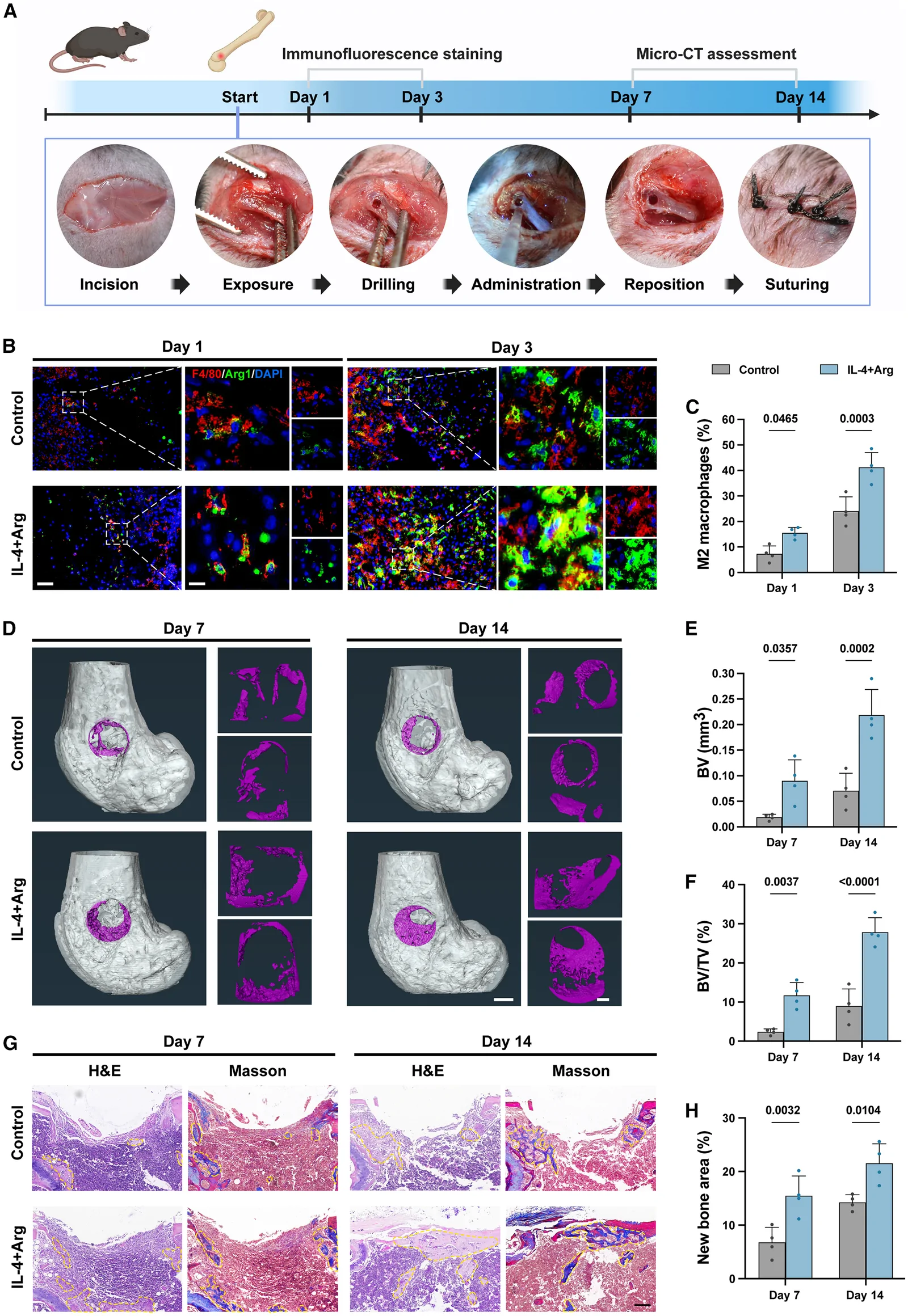
Simultaneous application of IL-4 and Arg promotes bone regeneration *in vivo* (A) Schematic illustration of the surgical procedure and timeline. (B and C) Evaluation of early macrophage polarization *in vivo*, showing representative immunofluorescence images of macrophages (F4/80, red) and M2 marker (Arg1, green) at days 1 and 3 post-surgery (B) and quantification of the percentage of M2 macrophages (Arg1^+^/F4/80^+^) (C). (D–F) Bone regeneration assessment via micro-CT, showing representative 3D reconstruction images of the bone defect area at day 7 and day 14 post-surgery (D) and quantification of BV (E) and BV/TV (F). (G and H) Histological evaluation of bone repair, showing representative images of H&E and Masson’s trichrome staining at day 7 and day 14 (G) and quantification of new bone area (%) (H). Error bars represent SD. Data were analyzed by two-way ANOVA followed by Sidak’s multiple comparisons test (C, E, F, and H). Sample sizes: *n* = 4 animals per group. Scale bars, 50 μm (B) and 10 μm (inset), 0.5 mm (D) and 200 μm (inset), 250 μm (G).

These data were further confirmed by longitudinal quantification of micro-CT and histological analyses 7 and 14 days after surgery (Figures 6D–6H). Indeed, three-dimensional reconstructions revealed the incremental enhancement of bone regeneration by the IL-4 + Arg treatment (Figures 6E and 6F), mirrored by more extensive new bone areas (outlined by dashed lines) and mature collagen deposition within the defect sites (Figures 6G and 6H).

In summary, these *in vivo* results validate the effectiveness of IL-4 + Arg application to enhance early bone regeneration by promoting sustained macrophage efferocytosis, which altogether highlights the translational potential of this strategy to stimulate bone regeneration following implant-associated injuries.

## Discussion

In this study, we established a synergistic IL-4 and Arg delivery strategy to optimize the immune-osteogenic microenvironment by enhancing macrophage efferocytosis, which may accelerate bone regeneration after implantation. We identified a critical spatiotemporal mismatch at the bone injury site: abundant ACs emerge early, whereas macrophage recruitment lags behind. The resulting accumulation of uncleared ACs induces oxidative stress, creating an inflammatory microenvironment that suppresses osteogenic differentiation of BMSCs. Mechanistically, IL-4 promotes macrophage polarization, whereas exogenous Arg sustains macrophage efferocytosis and Arg1-dependently facilitates the secretion of pro-reparative cytokines. Consequently, this coordinated intervention effectively transforms the pro-inflammatory, apoptotic niche into a pro-regenerative microenvironment, facilitating the initiation of osteogenesis and significantly enhancing early bone regeneration *in vivo*. Altogether, these findings underscore the translational potential of targeting macrophage efferocytosis to optimize the early immune microenvironment and accelerate new bone formation after implantation.

### Limitations of the study

This work primarily focused on the immunomodulatory and osteogenic-promoting effects of the IL-4 and Arg co-delivery strategy within the localized bone injury microenvironment. However, several limitations remain to be addressed. First, although our standardized femoral bone injury model successfully simulated clinical thermomechanical drilling trauma and early bone regeneration, it lacked a concurrent simulation of clinical implant surgery through implant placement. In realistic dental or orthopedic scenarios, the hydrogel matrix resides directly within the narrow interfacial gap between the host bone and the implant. Future studies should therefore optimize the delivery substrate to improve material retention after implant placement and appropriately control drug-release kinetics. Concurrently, true implant-placement animal models should be introduced and evaluated using pullout torque tests and longitudinal bone-to-implant contact (BIC) ratios to fully validate the translational stability of the system.

Second, although we evaluated the functional contribution of arginase metabolism *in vitro* using nor-NOHA, it should be noted that nor-NOHA acts as a pharmacological pan-arginase inhibitor with a preference toward Arg2 rather than as a specific Arg1 inhibitor. Therefore, potential secondary or off-target effects caused by the concurrent inhibition of both Arg1 and Arg2 cannot be entirely ruled out. Future validation should employ conditional gene deletion models, such as myeloid-specific Arg1 knockout *Arg1*^fl/fl^*Lyz2-CreERT2* mice, to fully elucidate the precise, cell-autonomous role of the Arg1 metabolic pathway. Additionally, our therapeutic efficacy was evaluated through early observations in healthy adult mice. We did not explore its long-term stability or validate its regenerative efficacy under compromised pathological bone beds, such as osteoporosis or skeletal aging. In senescent or osteoporotic skeletal microenvironments, aberrant cell death pathways can potently suppress endogenous bone regeneration, for instance, via the release of specialized necroptosis-derived extracellular vesicles that actively interfere with the directional differentiation of osteogenic lineages.^44^ Furthermore, systematically mapping the distinct transcriptomic, molecular, and epigenetic alterations of native BMSCs during skeletal aging is vital for expanding the generalizability of targeted biomaterial therapies to geriatric populations.^45^ Examining our efferocytosis-centered therapeutic paradigm in these chronic pathological models will be a major focus of our future investigations.

## Resource availability

### Lead contact

Requests for further information and resources should be directed to and will be fulfilled by the lead contact, Wenjie Zhang (zhangwenjie586@126.com).

### Materials availability

This study did not generate new unique reagents.

### Data and code availability

• The RNA-sequencing datasets generated in this study have been deposited in the Genome Sequence Archive (GSA) at the National Genomics Data Center (NGDC) under accession number GSA: CRA041253 and are publicly accessible.
• This paper does not report original code.
• Any additional information required to reanalyze the data reported in this paper is available from the lead contact upon request.

## Acknowledgments

This study was supported by the 10.13039/501100001809National Natural Science Foundation of China (grant no. 82370923), the 10.13039/501100012166National Key Research and Development Program of China (grant no. 2025YFC2426900), “Shuguang Program” supported by the 10.13039/501100003024Shanghai Education Development Foundation, and the 10.13039/501100003395Shanghai Municipal Education Commission (grant no. 25SG22). The graphical abstract and Figures 1, 2, 3, 4, 5, and 6 were partially created or adapted with BioRender.com. Fontana, T. (https://BioRender.com) licensed under CC BY 4.0.

## Author contributions

Conceptualization, L.M. and W.Z.; methodology, L.M., R.L., and S.W.; investigation, L.M., R.L., S.W., Junyi Wang, R.Y., X.S., H.Z., and Jiajia Wang; formal analysis, L.M., R.L., and S.W.; writing – original draft, L.M.; writing – review and editing, L.M., I.A.V., and W.Z.; visualization, L.M.; funding acquisition, W.Z.; resources, W.Z.; supervision, W.Z.

## Declaration of interests

The authors declare no competing interests.

## STAR★Methods

### Key resources table

**Table:** 

REAGENT or RESOURCE	SOURCE	IDENTIFIER
**Antibodies**		

Anti-F4/80 antibody	Abcam	Cat# ab90247; RRID:AB_10712189
Anti-iNOS antibody	Abcam	Cat# ab178945; RRID:AB_2861417
Anti-Arginase 1 antibody	Cell Signaling Technology	Cat# 93668; RRID:AB_2800207

**Chemicals, peptides, and recombinant proteins**		

Gelatin methacryloyl (GelMA)	Engineering For Life Co., Ltd	EFL-GM-60
Recombinant murine IL-4	PeproTech	Cat# 214–14
L-Arginine (L-Arg)	Aladdin	Cat# A103485
Macrophage colony stimulating factor (M-CSF)	PeproTech	Cat# 315–02
Nor-NOHA Acetate	Selleck	Cat# S9737

**Critical commercial assays**		

Mouse TGF-β1 ELISA Kit	ABclonal	Cat# RK00057
Mouse IL-10 ELISA Kit	Elabscience	Cat# E-EL-M0046
Arg Content Assay Kit	Solarbio	Cat# BC5635
Mouse IL-4 ELISA Kit	Elabscience	Cat# E-EL-M0043
TUNEL Apoptosis Detection Kit	Yeasen	Cat# 40308

**Deposited data**		

RNA-seq	This paper	GSA: CRA041253

**Experimental models: Cell lines**		

MC3T3-E1 (Mouse pre-osteoblast cell line)	Shanghai Institutes for Biological Sciences	Cat# SCSP-5218
RAW 264.7 (Murine macrophage cell line)	Procell Life Science and Technology Co., Ltd.	Cat# CL-0190

**Experimental models: Organisms/strains**		

C57BL/6 mice	Shanghai Laboratory Animal Center	N/A

**Software and algorithms**		

GraphPad Prism 10 software	GraphPad Prism	RRID:SCR_002798
ImageJ software	National Institutes of Health	RRID:SCR_003070
FlowJo V10 software	FlowJo, LLC	RRID:SCR_008520
Avizo 3D Software	Thermo Fisher Scientific	RRID:SCR_014431

### Experimental model and study participant details

#### Cell culture

The mouse pre-osteoblast cell line MC3T3-E1 (Shanghai Institutes for Biological Sciences, China) was cultured in α minimum essential medium (α-MEM, HyClone, Logan, UT, USA) containing 10% fetal bovine serum (FBS, Gibco, Thermo Fisher Scientific, Waltham, MA, USA) and 1% penicillin-streptomycin. The murine macrophage cell line RAW 264.7 was obtained from Procell Life Science and Technology Co., Ltd. (Wuhan, China). These cells were maintained in high-glucose Dulbecco’s Modified Eagle Medium (DMEM, HyClone) supplemented with 10% FBS and 1% penicillin-streptomycin. The cells were cultured in a 5% CO_2_ incubator at 37 °C. The identities of the MC3T3-E1 and RAW264.7 cell lines were confirmed by STR profiling. Both cell lines tested negative for mycoplasma contamination.

BMSCs were isolated from the femurs and tibias of 4-week-old C57BL/6 mice. Briefly, bone marrow plugs were flushed out and resuspended in DMEM containing 10% FBS. Cells were incubated at 37 °C in a humidified atmosphere of 5% CO_2_. Non-adherent cells were removed after 24 h, and the medium was refreshed every 3 days. BMSCs at passages 2–3 were used for *in vitro* osteogenic experiments.

BMDMs were differentiated from bone marrow cells as previously described. Briefly, bone marrow cells were flushed from femurs and cultured in RPMI-1640 medium (Gibco) supplemented with 10% FBS and 20 ng/mL macrophage colony stimulating factor (M-CSF, PeproTech, Rocky Hill, NJ, USA). The cells were cultured in a 5% CO_2_ incubator at 37 °C. Half of the induction medium was refreshed on day 3, and mature BMDMs were harvested on day 5 for subsequent experiments.

#### Construction of mouse model

All animal experiments were approved by the Animal Care and Use Committee of Ninth People’s Hospital, Shanghai Jiao Tong University School of Medicine (Approval number: SH9H-2020- A227-1) and performed in strict accordance with the institutional guidelines for the care and use of laboratory animals. Male wild-type C57BL/6 mice were purchased from Shanghai Laboratory Animal Center. 4-week-old male mice (weighing 12–17 g) were utilized for the primary isolation and harvest of BMSCs. 8-week-old male mice (weighing 20–25 g) were utilized for the *in vivo* animal models, including both the femoral defect and calvarial injury models. All animals were maintained in a specific pathogen-free (SPF) facility under standardized environmental conditions (temperature: 22°C–24°C, humidity: 50–60%, 12-h light/dark cycle) with free access to standard laboratory chow and water. Only male mice were chosen in this study to minimize potential estrogenic fluctuations on systemic bone metabolism and inflammation.

For the calvarial injury model, the parietal bone was exposed, and a 2 mm long non-penetrating linear defect was created using a spherical burr. The incision was sutured, and mice were sacrificed at 12 h, 24 h, 48 h, and 72 h post-surgery for sample collection. For the femoral defect model, the distal femur was exposed under general anesthesia. A cylindrical defect (1 mm in diameter, 1.2 mm in depth) was created using a dental spherical burr, with intermittent physiological saline irrigation to prevent heat-induced damage. The defects were filled with either Control GelMA hydrogel or IL-4 + Arg loaded GelMA hydrogel and photocrosslinked *in situ*. Following layer-by-layer suturing, mice were sacrificed at 1, 3, 7, and 14 days post-surgery for analysis.

### Method details

#### Establishment of *in vitro* apoptosis model

To mimic the cellular damage caused by implant site preparation (drilling) *in vitro*, MC3T3-E1 were subjected to mechanical and thermal injuries. Cells were seeded in culture plates and allowed to adhere overnight under standard conditions (37 °C, 5% CO_2_).

For the mechanical injury group, cells were subjected to physical scraping using a sterile pipette tip to simulate friction damage. For the thermal injury group, cells were exposed to heat shock at 47 °C for 1 min in a water bath to simulate frictional heat. For the combined injury group, cells received both mechanical stimulation and thermal shock sequentially. Following the injury procedures, apoptosis was confirmed using an Annexin V-APC/PI Apoptosis Detection Kit (KeyGEN BioTECH, Nanjing, China) via flow cytometry. Untreated cells cultured under identical conditions served as the negative control.

#### Cell viability and proliferation assays

The cell viability of different groups was evaluated with Cell Counting Kit 8 (CCK-8, Beyotime). Cells were seeded in 96-well plates. At 24, 48, and 72 h, the culture medium was replaced with fresh medium containing 10% CCK-8 solution. After incubation at 37 °C for 2 h, the absorbance of the solution at 450 nm was measured with a microplate spectrophotometer.

Cell proliferation was observed by laser confocal microscope at 24 h, 48 h, 72 h. Briefly, the proliferation of cells was assessed by BeyoClick EdU-488 Cell Proliferation Kit (Beyotime) according to the manufacturer’s instructions. The samples were incubated with 10 μM EdU for 2 h, fixed with 4% paraformaldehyde and stained with the Apollo reaction cocktail according to the manufacturer’s protocol. Nuclei were counterstained with DAPI (Beyotime) for 10 min. All staining was performed at room temperature in the dark. Finally, the fluorescence images of the stained cells were obtained using a confocal laser scanning microscope (Leica TCS SP8, Leica Microsystems, Wetzlar, Germany) and analyzed with ImageJ software.

#### Cell migration assay

For the cell transwell assay, 24-well Transwell inserts (8.0 μm pore size, LABSELECT, Beijing, China) were utilized to evaluate the recruitment effect of the synergistic strategy. Macrophages were suspended in serum-free medium and seeded into the upper chamber at a density of 5 × 10^4^ cells/well. The lower chamber was filled with complete medium containing different inducers: Control, IL-4, Arg, or IL-4 + Arg. After 48 h of incubation in the cell incubator, non-migrated cells on the upper surface of the membrane were gently removed with a cotton swab. The cells that had migrated to the lower surface of the membrane were fixed with 4% paraformaldehyde for 15 min and stained with 0.1% crystal violet (Solarbio, Beijing, China) for 30 min. Migrated cells were imaged using an inverted microscope, and the cell number was quantified in five random fields per well using ImageJ software.

For the wound-healing assay, BMSCs and MC3T3-E1 cells (5 × 10^4^ cells/mL) were seeded in 24-well plates and cultured until reaching 90% confluence. A linear wound was created by scratching the cell monolayer with a sterile 200 μL pipette tip. The cells were washed with PBS to remove debris and subsequently cultured in the conditional media prepared as described above. Images of the wound area were captured at 0 h and 24 h using an optical microscope. The wound closure rate was calculated using ImageJ software based on the change in wound area.

#### Macrophage polarization and efferocytosis assay

For macrophage polarization, cells were cultured in complete medium supplemented with 10% FBS at 37 °C in a 5% CO_2_ incubator. To induce M2 polarization, macrophages were treated with 20 ng/mL recombinant murine IL-4 (PeproTech) and 2 mM L-Arg (Aladdin) for 24 h.^46^^,^^47^

Efferocytosis capacity was assessed by quantifying the uptake of ACs by macrophages *in vitro*. Briefly, macrophages were seeded onto culture plates and allowed to adhere overnight. To distinguish between the two cell populations, macrophages were labeled with CellTracker Green CMFDA (Yeasen), while ACs were generated as described above and subsequently labeled with CellTracker CM-Dil (Yeasen). Labeled ACs were then added to macrophages at a ratio of 5:1 (ACs:Macrophages) and co-cultured at 37 °C for indicated time points (6 h, 24 h, and 48 h). After incubation, non-engulfed ACs were removed by gentle washing with PBS. For quantitative analysis, macrophages were harvested, and efferocytosis efficiency was analyzed by flow cytometry based on the percentage of double-positive cells. For morphological observation, cells were fixed with 4% paraformaldehyde and imaged using a confocal microscope. Additionally, nuclei were counterstained with DAPI. Untreated macrophages incubated without ACs served as a negative control.

#### Flow cytometry analysis

Flow cytometry was employed for apoptosis detection and quantification of macrophage efferocytosis efficiency. For both assays, cells were harvested by gentle trypsinization (EDTA-free for apoptosis assay), washed twice with cold PBS, and analyzed using a flow cytometer (BD FACSAria III, BD Biosciences, Inc., San Jose, USA). Data analysis was performed using FlowJo V10 software (FlowJo, LLC, Ashland, OR, USA).

For the detection of apoptosis following mechanical and thermal injuries, an Annexin V-APC/PI Apoptosis Detection Kit (KeyGEN BioTECH) was utilized. The harvested cells were resuspended in 500 μL of 1 × Binding Buffer containing 5 μL of Annexin V-APC and 5 μL of Propidium Iodide (PI). After incubation at room temperature for 15 min in the dark, cell populations were gated to distinguish between viable (Q4), early apoptotic (Q3), late apoptotic (Q2), and necrotic cells (Q1).

For the quantification of efferocytosis efficiency, macrophages (labeled with CMFDA) co-cultured with ACs (labeled with CM-Dil) were collected. Efferocytosis efficiency was determined by calculating the percentage of phagocytic cells, defined as the proportion of macrophages that had engulfed at least one AC. Macrophages were identified by their green fluorescence (CMFDA), and engulfed ACs were identified by red fluorescence (CM-Dil). Thus, phagocytosis was characterized by the detection of double-positive (CMFDA^+^/CM-Dil^+^) cells. The efficiency was calculated using the following formula:

$$\text{Phagocytosis}\;\left(\%\right)=\frac{\text{Number}\;\text{of}\;{\text{CMFDA}}^{+}/{\text{CM}-\text{Dil}}^{+}\;\text{Cells}}{\text{Number}\;\text{of}\;{\text{CMFDA}}^{+}\;\text{Cells}}\times 100\%$$

#### ROS measurement

To evaluate the oxidative stress levels in ACs and the subsequent microenvironmental remodeling following the efferocytosis process, an ROS assay kit based on the DCFH-DA fluorescent probe was utilized. Briefly, the cells were incubated with 10 μM DCFH-DA working solution in serum-free medium at 37 °C for 30 min in the dark. For *in situ* observation, nuclei were counterstained with DAPI, and images were captured using fluorescence microscopy. Additionally, the relative fluorescence intensity was quantified using a microplate reader to assess intracellular ROS accumulation and evaluate the therapeutic efficacy of the synergistic IL-4 and Arg strategy in improving the pro-regenerative microenvironment.

#### ELISA assay

The secretion levels of anti-inflammatory cytokines were quantified using a Mouse TGF-β1 ELISA Kit (ABclonal, Wuhan, China) and a Mouse IL-10 ELISA Kit (Elabscience, Wuhan, China). Briefly, after treating macrophages for 48 h, the cell supernatants were collected and centrifuged to remove particulates. According to the manufacturer’s instructions, 100 μL of the standard solution or sample was added to a 96-well plate from the kit and placed at 37 °C for 120 min. Following six washes with the provided washing buffer, the specific biotinylated detection antibody was added and incubated at 37 °C for 1 h. The plates were washed again, and 100 μL of streptavidin horseradish peroxidase reagent was added and incubated for another 30 min. After washing six times, 100 μL of a Streptavidin-Horseradish Peroxidase (HRP) working solution was added for a 30-min incubation. After the final washing steps, 100 μL of substrate reagent was added to each well, and the plates were incubated for 15 min at 37 °C in the dark. The reaction was terminated by adding stop solution, and the OD was measured immediately at 450 nm using a microplate reader.

#### RNA isolation and qRT-PCR

Total RNA was extracted from cell samples using an RNA extraction kit (ESSience, Shanghai, China) according to the manufacturer’s instructions. The concentration and purity of RNA were verified using a spectrophotometer (NanoDrop, Thermo Fisher Scientific). Subsequently, complementary DNA (cDNA) was synthesized using Hifair III 1st Strand cDNA Synthesis SuperMix (Yeasen, Shanghai, China). qRT-PCR was performed using Hieff qPCR SYBR Green Master Mix (YEASEN) on a LightCycler 480 II Real-Time PCR System (Roche Diagnostics, Rotkreuz, Switzerland). The cycling conditions were set as follows: initial denaturation at 95 °C for 3 min, followed by 40 cycles of 95 °C for 10 s and 60 °C for 1 min. GAPDH was used as the housekeeping gene for normalization. Relative gene expression was calculated using the 2^−ΔΔCt^ method, and data were analyzed using GraphPad Prism 10 software. The primer sequences are listed in Table S1.

#### RNA sequencing and bioinformatics analysis

Total RNA was extracted from BMDMs for library construction and transcriptomic sequencing. Bioinformatics analysis was performed using the Majorbio Cloud Platform. DEGs between groups were identified using the DESeq2 algorithm, with the significance threshold set at an adjusted *p*-value ≤0.05. Volcano plots and hierarchical clustering heatmaps were generated to visualize global gene expression patterns and identify key regulatory targets. Functional annotation and pathway enrichment were conducted via GO, KEGG, and Gene Set Enrichment Analysis (GSEA) to explore biological processes and signaling pathways related to efferocytosis, Arg metabolism, and anti-inflammatory responses.

#### *In vitro* arginase inhibition

To experimentally verify the dependence of the synergistic therapeutic paradigm on the Arg1 metabolic pathway, a pharmacological blockade assay was performed. BMDMs were pre-treated with 50 μM of nor-NOHA (Selleck, Houston, TX, USA). The cells were then co-stimulated with the combination of 20 ng/mL IL-4 and 2 mM Arg in the presence or absence of nor-NOHA. After 24 h of incubation, cells were harvested for total RNA extraction and subsequent qRT-PCR analysis to evaluate the transcriptional levels of *Tgfb1* and *Il10*. Concurrently, after 48 h of treatment, the culture supernatants were collected and centrifuged at 2,000 rpm for 10 min to remove cellular debris, followed by ELISA quantification to determine the secreted protein concentrations of TGF-β1 and IL-10.

#### Conditioned media preparation and osteogenic differentiation

Macrophages were seeded and subjected to the different treatments: Control group (conditioned medium from untreated macrophages), IL-4 + Arg group (conditioned medium from macrophages stimulated with IL-4 and Arg), Efferocytosis group (conditioned medium from macrophages co-cultured with ACs), and IL-4 + Arg & Efferocytosis group (conditioned medium from macrophages treated with IL-4 and Arg in the presence of ACs). The supernatants were collected after 24 h of incubation. The collected supernatants were centrifuged at 2,000 rpm for 10 min to remove cell debris and mixed with fresh complete α-MEM at a 1:1 ratio to generate the final conditional media.

MC3T3-E1 cells and BMSCs were seeded and cultured in the prepared conditioned media. To induce osteogenic differentiation, the conditional media were supplemented with 50 μg/mL ascorbic acid (Sigma-Aldrich, St. Louis, MO, USA), 10 nM dexamethasone (Sigma-Aldrich), and 10 mM β-glycerophosphate (Sigma-Aldrich). The medium was changed every 2–3 days.

For ALP activity assays, cells were cultured in 12-well plates for 7 days, and subsequently fixed with 4% paraformaldehyde and stained with the BCIP/NBT Alkaline Phosphatase Color Development Kit (Beyotime, Shanghai, China). For ALP quantitative assay, the cells were incubated with *p*-nitrophenyl phosphate (*p*NPP) (Sigma, St. Louis, MO, USA). Total protein content was calculated using the Bio-Rad protein assay kit (Bio-Rad, USA) and normalized with a series of BSA (Sigma) standard at 630 nm. ALP activity was expressed as the optical density (OD) values at 405 nm per milligram of total proteins. Images were captured using an inverted phase contrast microscope (Olympus IX70, Tokyo, Japan) and analyzed using the ImageJ software.

#### *In vivo* sequential fluorescent labeling

To dynamically track early osteogenic priming and the process of new bone formation following injury, an *in vivo* sequential fluorescent labeling protocol was implemented. Mice were intraperitoneally injected with Alizarin Red S (30 mg/kg) on day 4 and Calcein (20 mg/kg) on day 6 post-surgery. On day 9, mice were sacrificed, and the bone specimens were harvested and fixed in 4% paraformaldehyde. Following gradient ethanol dehydration and xylene clearing, the samples were embedded in polymethyl methacrylate (PMMA). Hard-tissue sections (50 μm final thickness) were prepared using a high-precision microtome and imaged via confocal laser scanning microscopy to evaluate mineral apposition and early bone regeneration at the injury site.

#### Preparation of Arg/IL-4-loaded GelMA hydrogel

Gelatin methacryloyl (GelMA) and lithium phenyl-2,4,6-trimethylbenzoylphosphinate (LAP) were purchased from Engineering for Life Co., Ltd (Suzhou, China). To prepare the hydrogel precursor solution, lyophilized GelMA was dissolved in phosphate-buffered saline (PBS) at 50 °C under constant stirring to obtain a homogeneous 5% (w/v) solution. The photoinitiator LAP was added to the solution at a final concentration of 0.25% (w/v). For the fabrication of therapeutic hydrogels, Arg and recombinant murine IL-4 were incorporated into the precursor solution to achieve final concentrations of 20 mM and 200 ng/mL. The mixture was then transferred into molds and photocrosslinked using a 405 nm light source for 30 s to form the hydrogels.

#### Characterization of Arg/IL-4-loaded GelMA hydrogel

To characterize the injectability of the hydrogel system, the handling performance and fluidic behavior of the prepared GelMA hydrogel precursor solution were evaluated by extruding it through a standard pipette tip. Additionally, the precursor solution was loaded into a syringe and injected directly into an aqueous environment via a fine syringe needle for observation. Photographs were captured for the aforementioned steps.

Rheological characterization of the hydrogel precursor solution was performed using a rotational rheometer at room temperature. To evaluate its specific fluid properties and identify typical shear-thinning behavior, the steady-state shear viscosity was monitored and plotted on a log-log scale as a function of an increasing shear rate.

For the *in vitro* degradation assay, the photo-crosslinked therapeutic GelMA hydrogels were fabricated and their initial weights (W_0_) were precisely recorded. Subsequently, the hydrogel samples were completely immersed in PBS (pH 7.4) and continuously incubated under simulated physiological conditions at 37°C. At scheduled daily intervals over a 7-day incubation period, the hydrogels were retrieved, gently blotted with filter paper to thoroughly remove excess surface liquid, and weighed to determine the remaining weight (W_t_). The chronological mass loss profile was tracked by calculating the weight remaining ratio according to the following formula:

$$\text{Weight}\;\text{remaining}\;\text{ratio}\;\left(\%\right)=\left(\frac{W_{t}}{W_{0}}\right)\times 100\%$$

To evaluate drug release kinetics, the drug-loaded hydrogels were incubated in PBS at 37 °C. At scheduled time points over 14 days, supernatants were collected and replaced with fresh PBS. The cumulative release of Arg was quantified using an Arg Content Assay Kit (Solarbio, Beijing, China), and the release of IL-4 was determined using a Mouse IL-4 ELISA Kit (Elabscience, Wuhan, China). Additionally, the biocompatibility of the hydrogels was assessed by performing Live/Dead staining using a Calcein AM/PI Double Stain Kit (Servicebio, Wuhan, China) after 3 days of culture.

#### Micro-CT imaging and analysis

The fixed femoral specimens were examined and imaged using Micro-CT (Skyscan 1276, Bruker, Kontich, Belgium) to evaluate the newly formed bone within the defect area. The scanning parameters were set at 80 kV, an exposure time of 3,000 ms, and a scanning pixel size of 5 μm. After scanning, 3D visualization and analysis were performed using Avizo software (Version 2022.1, Thermo Fisher Scientific, Waltham, MA, USA). The region of interest (ROI) corresponding to the original bone defect was defined, and the newly formed bone was segmented from the surrounding tissue. Quantitative morphometric analysis was performed to calculate the BV and BV/TV.

#### Histological and immunofluorescence analysis

The femurs (*n* = 4 per group) were fixed in 4% paraformaldehyde, decalcified in 10% EDTA for 2 weeks, and embedded in paraffin wax. A series of 5 μm sections was sliced from the defect region. To evaluate new bone formation and collagen maturation, sections were stained with H&E and Masson’s Trichrome, respectively. Images were captured using an optical microscope, and the area of newly formed bone was quantified using ImageJ software.

For immunofluorescence analysis, decalcified sections were subjected to antigen retrieval and blocked with 5% normal goat serum for 1 h at room temperature. The sections were then incubated overnight at 4 °C with primary antibodies against F4/80 (1:200, Cat. No. ab90247, Abcam, Cambridge, UK), iNOS (1:50, Cat. No. ab178945, Abcam) and Arg1 (1:50, Cat. No. 93668, Cell Signaling Technology, Danvers, MA, USA) to identify macrophages and their M2 polarization status. Following three washes with PBS, the sections were incubated with corresponding fluorophore-conjugated secondary antibodies for 1 h at room temperature in the dark. To assess cell apoptosis *in situ*, a TUNEL Apoptosis Detection Kit (Alexa Fluor 640, Yeasen, Shanghai, China) was employed according to the manufacturer’s instructions. Cellular nuclei were counterstained with DAPI. Fluorescence images were captured using a confocal laser scanning microscope (Leica TCS SP8). The number of positive cells in the defect region was quantified using ImageJ software.

### Quantification and statistical analysis

All quantitative data are presented as mean ± standard deviation (SD). The detailed statistical parameters for each individual experiment are explicitly indicated in the respective figure legends. Statistical analyses were performed using GraphPad Prism 10 software (GraphPad Software, Boston, MA, USA). For comparisons between two groups, an unpaired two-tailed Student’s *t* test was used. For comparisons among multiple groups, a one-way analysis of variance (ANOVA) followed by Tukey’s post hoc test was performed. For multi-variable datasets involving multiple time points and concentrations, a two-way ANOVA followed by Sidak’s multiple comparisons test was conducted. A *p*-value <0.05 was considered statistically significant. All experiments were repeated at least three times (*n* ≥ 3) as indicated in the figure legends.
