# Supplementary material for: IL-4 and arginine synergy accelerates early bone regeneration via enhanced efferocytosis and ameliorated microenvironment
Source: iScience. 2026 Sep 22;29(10):117739. doi: 10.1016/j.isci.2026.117739 (PMC13657358; doi:10.1016/j.isci.2026.117739)
Supplement: Supplementary file 1 [file mmc1.pdf]

iScience, Volume *29*

Supplemental information

IL-4 and arginine synergy accelerates early bone regeneration via enhanced efferocytosis and ameliorated microenvironment

Lingxi Meng, Rongpu Liu, Siyi Wang, Junyi Wang, Ilya A. Vinnikov, Rong Yang, Xinyu Song, Hongming Zhang, Jiajia Wang, and Wenjie Zhang

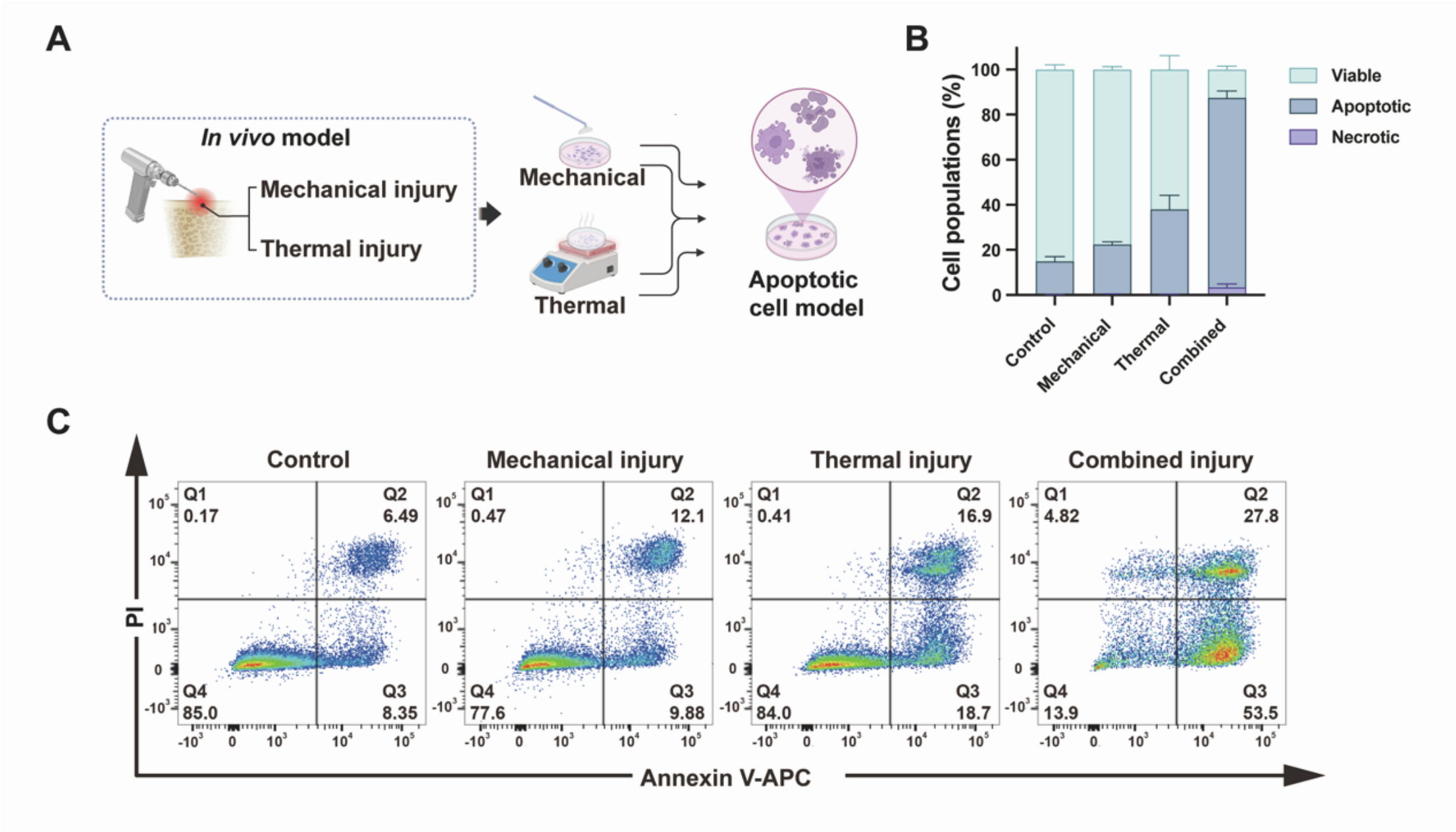

Figure S1. Establishment and characterization of the *in vitro* apoptotic cell model. Related to Figure 1.

(A) Schematic diagram of the establishment of *in vitro* injury models simulating the mechanical and thermal damages observed in the *in vivo* bone drilling process.

(B) Quantitative analysis of viable, apoptotic (Annexin V positive), and necrotic (PI positive) MC3T3-E1 populations after different treatments. Data are presented as mean ± SD (n = 3 biological replicates, each performed with 3 technical replicates).

(C) Representative flow cytometry plots of cells stained with Annexin V-APC and PI, demonstrating the distribution of cell populations across control, mechanical, thermal, and combined injury groups. n = 3 biological replicates.

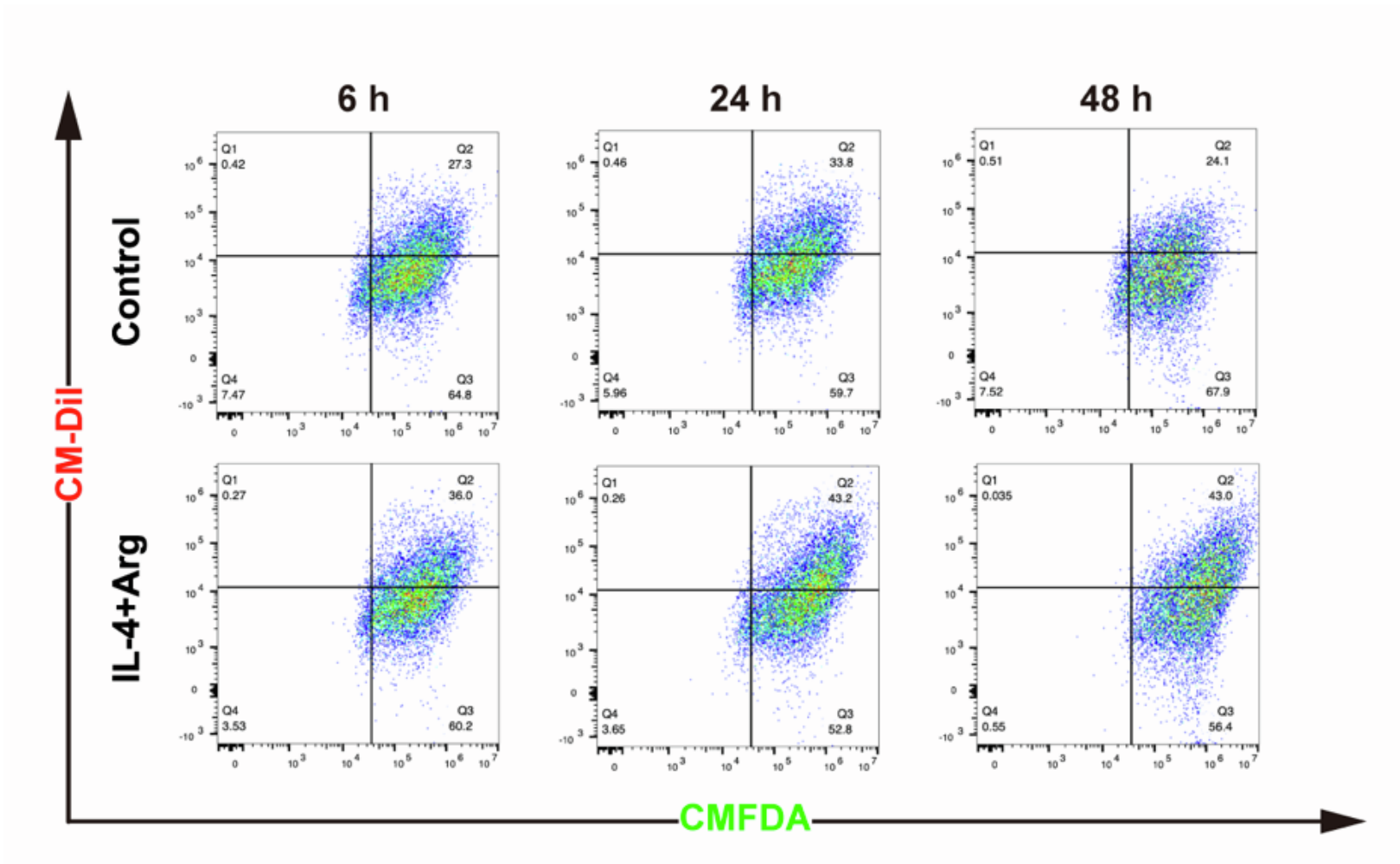

Figure S2. Flow cytometric evaluation of *in vitro* macrophage efferocytosis capacity at different time points. Related to Figure 3.

Representative flow cytometry scatter plots demonstrating the engulfment of CM-Dil-labeled apoptotic MC3T3-E1 cells (red) by CMFDA-labeled macrophages (green) at different incubation time points (6, 24, and 48 h) under Control and IL-4+Arg synergistic treatment conditions. The upper-right quadrant (Q2) represents the double-positive ($CMFDA^+$/$CM\text{-}Dil^+$) cell population, with its percentage corresponding to the successfully executed efferocytosis efficiency. *n* = 3 independent biological replicates.

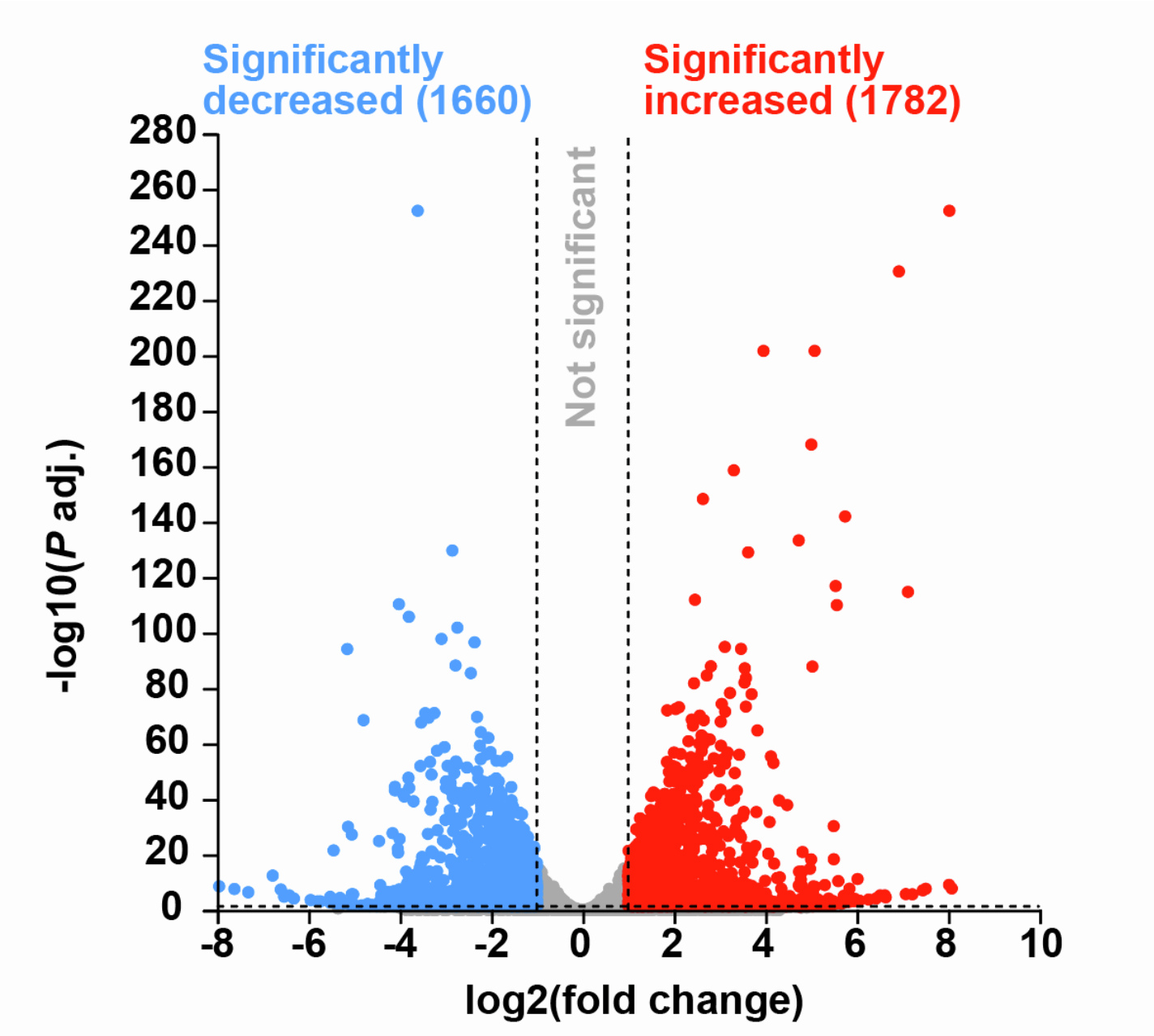

Figure S3. Volcano plot of differentially expressed genes in macrophages. Related to Figure 4.

Volcano plot displaying the distribution of DEGs between the IL-4 + Arg treatment group and the IL-4 induced group. Red dots represent significantly upregulated genes, while blue dots represent significantly downregulated genes. *n* = 3 biological replicates.

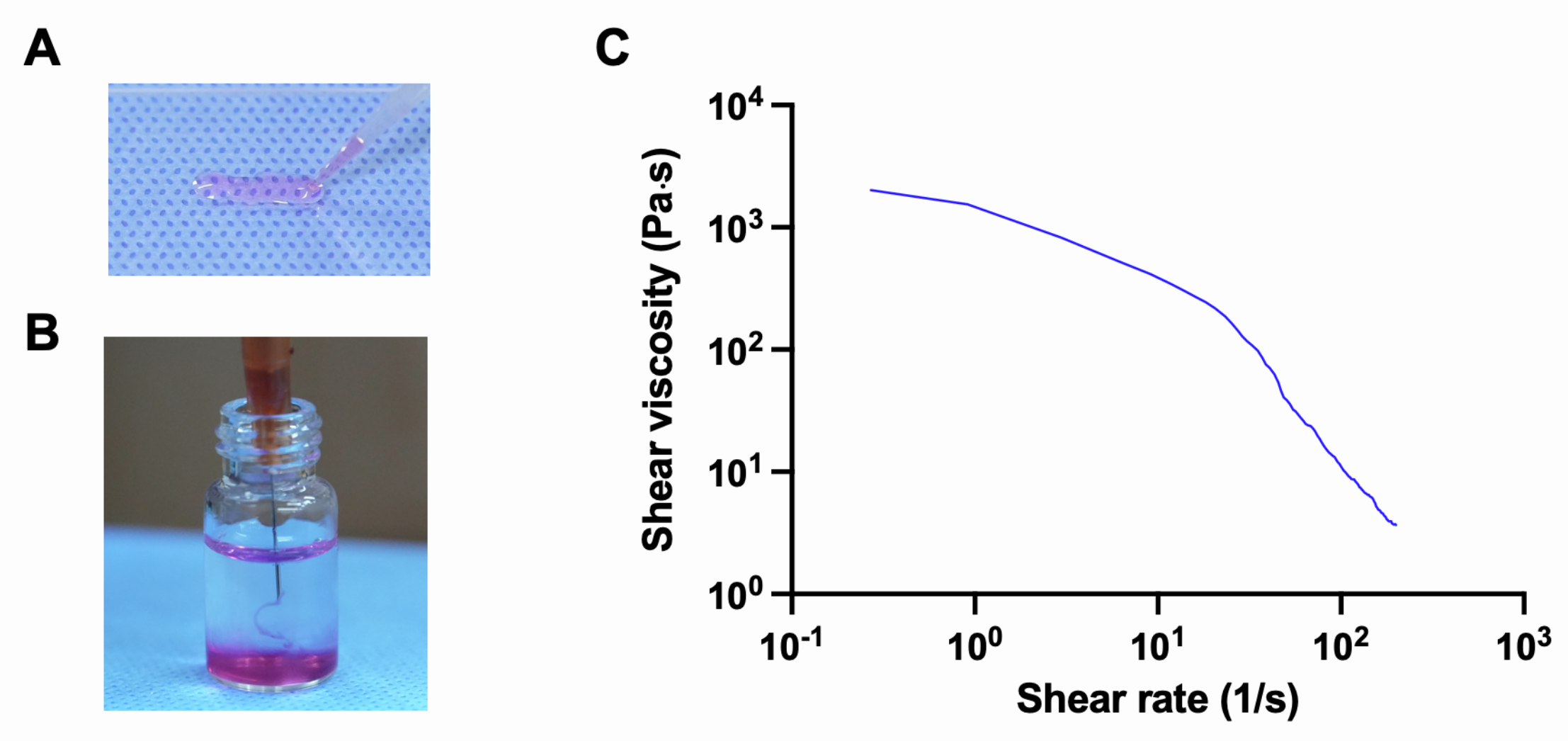

Figure S4. Injectability characterization of the GelMA hydrogel system. Related to Figure 6.

(A) Photograph illustrating the excellent handling performance and fluidic behavior of the prepared GelMA hydrogel precursor solution extruded through a pipette tip.

(B) Image demonstrating the injectability of the hydrogel, forming continuous fibrous filaments when directly injected into an aqueous environment via a fine syringe needle.

(C) Rheological profile showing the shear viscosity with a shear rate ranging from approximately 0.3 s^{-1} to 200 s^{-1} at 37 °C.

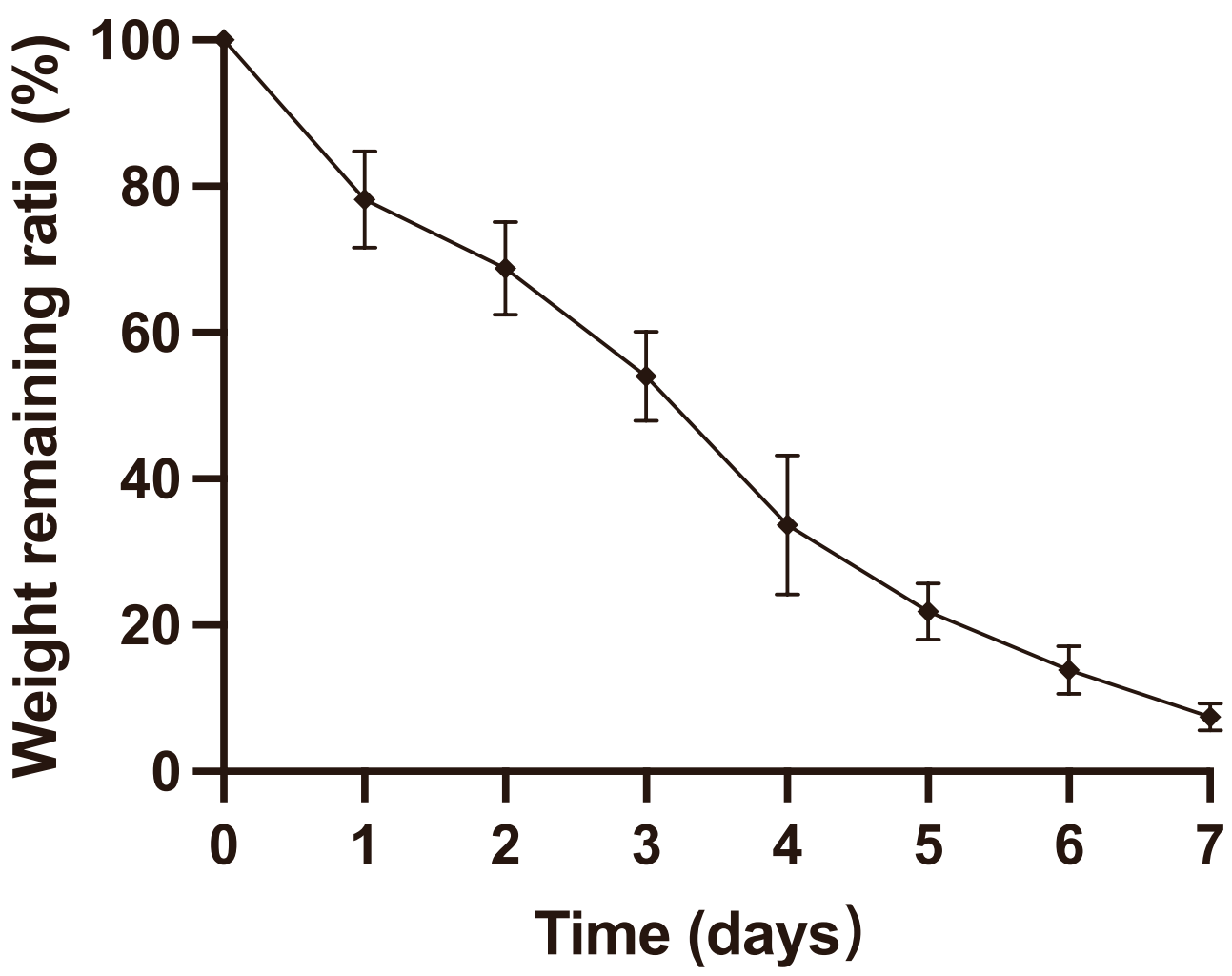

Figure S5. *In vitro* degradation profile of the GelMA hydrogel over time. Related to Figure 6.

Quantitative cumulative weight loss curve tracking the remaining weight ratio (%) of the photo-crosslinked therapeutic GelMA hydrogel during continuous incubation under physiological conditions (PBS, pH 7.4) at 37 °C over 7 days. Data are presented as mean ± SD (n = 4 independent experimental replicates).

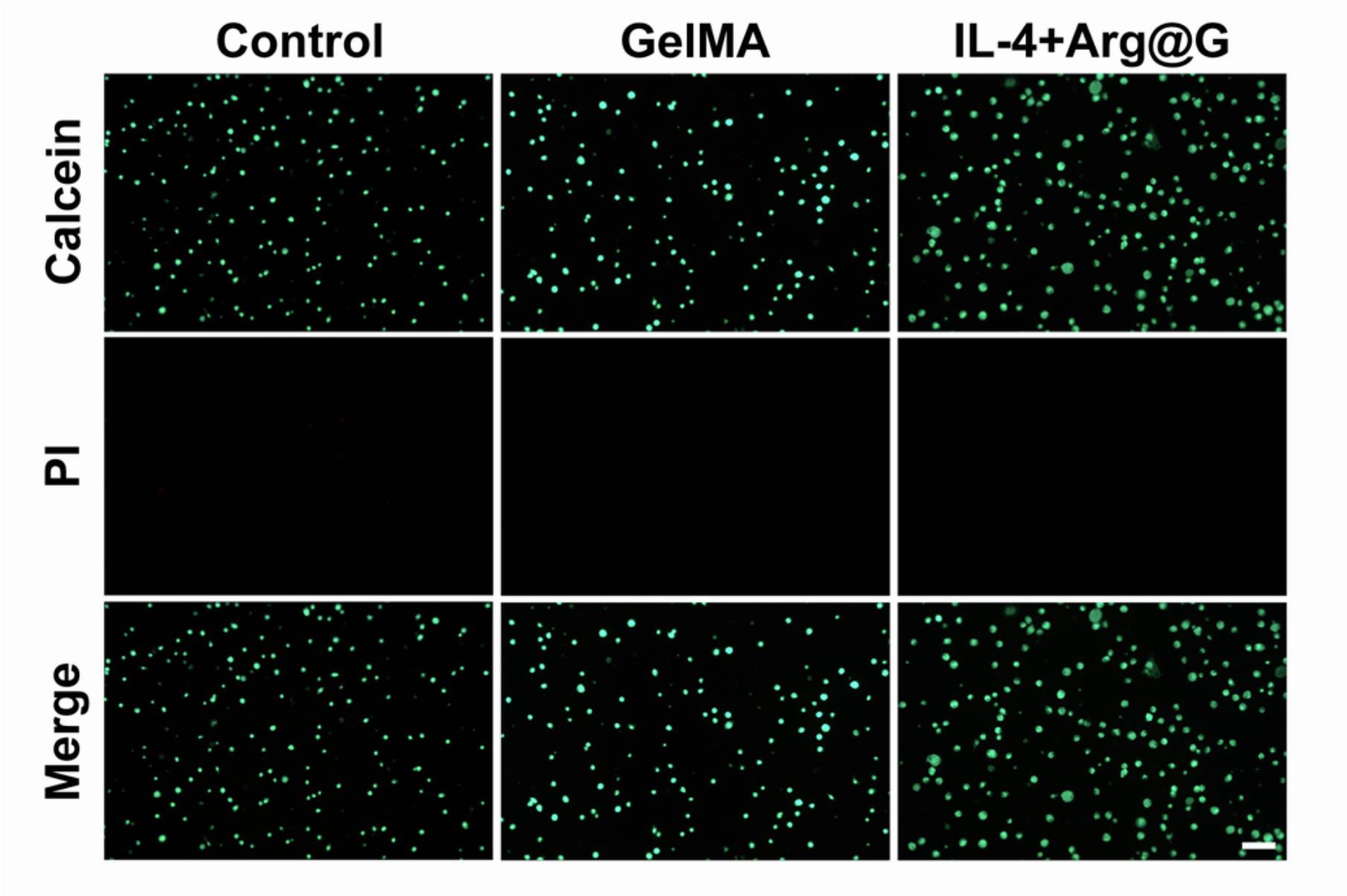

Figure S6. Biocompatibility evaluation of the hydrogel system. Related to Figure 6. Representative Live/Dead staining images of macrophages co-cultured with hydrogels, where live cells are stained with Calcein (green) and dead cells with PI (red). Scale bar = 100 μm, *n* = 3 biological replicates.

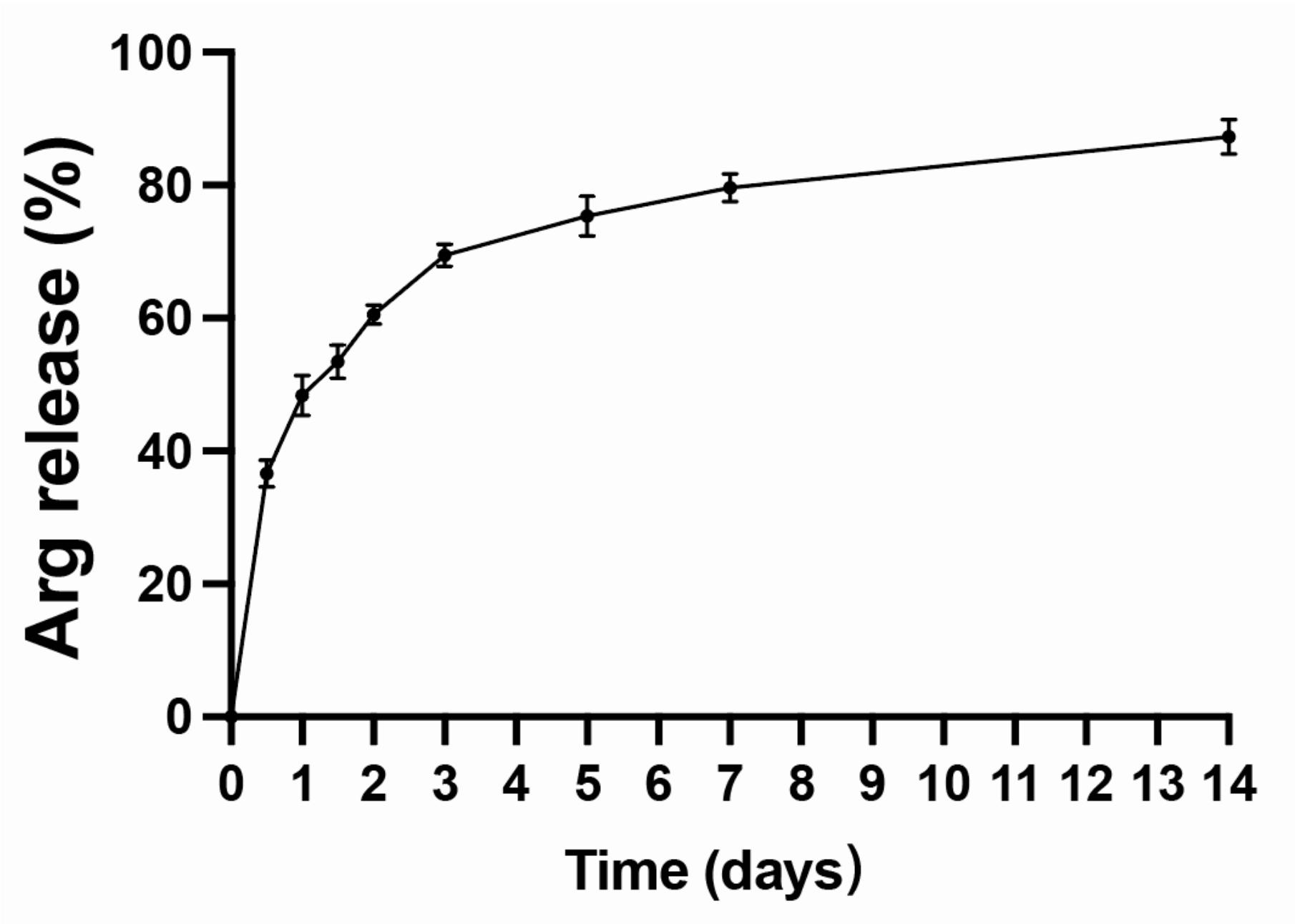

Figure S7. ***In vitro*** **release profile of Arginine. Related to Figure 6.**

Cumulative release profile of Arg from the GelMA hydrogel over 14 days. Data are presented as mean ± SD (n = 4 independent replicates).

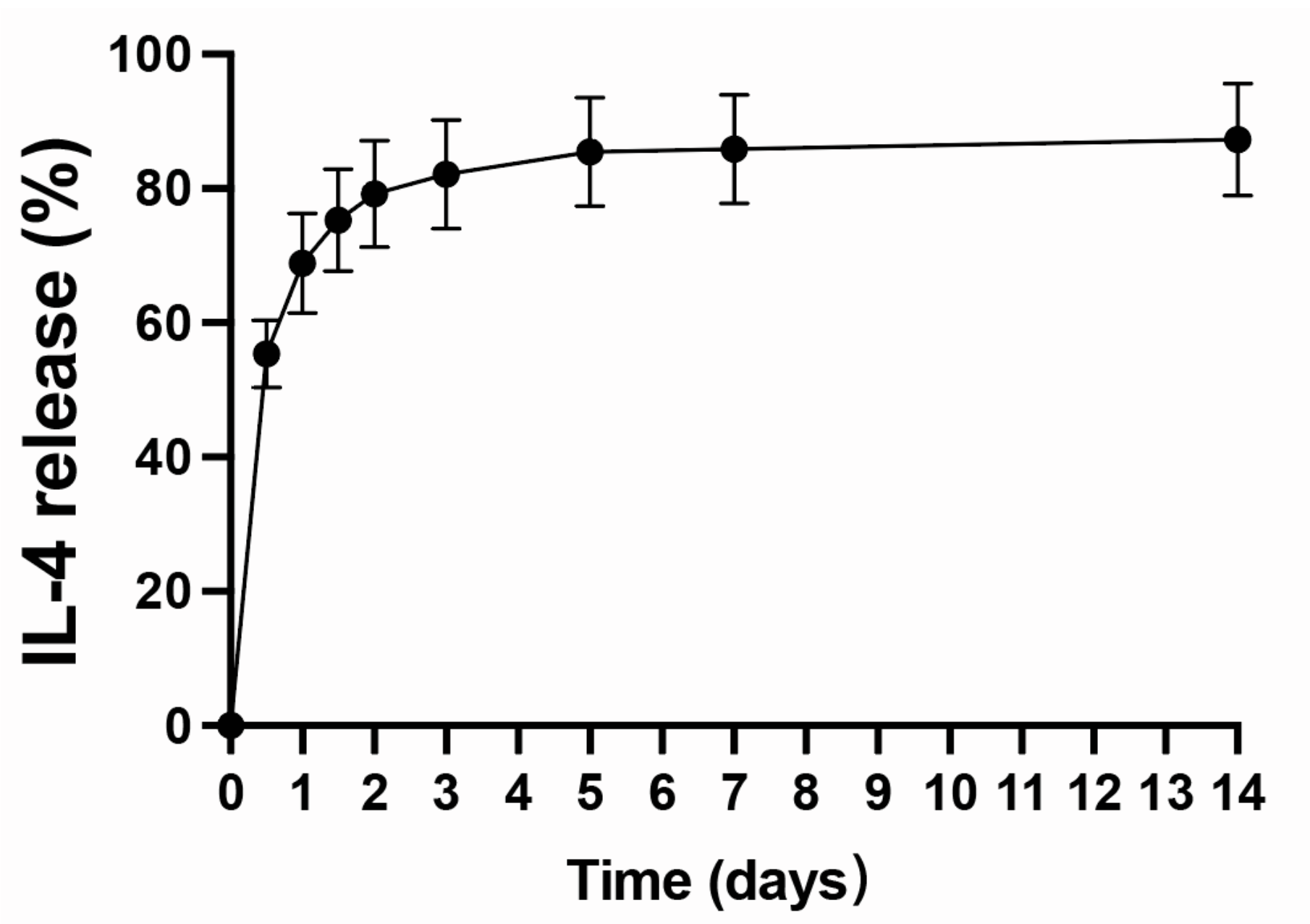

Figure S8. ***In vitro*** **release profile of IL-4. Related to Figure 6.**

Cumulative release profile of IL-4 from the GelMA hydrogel over 14 days. Data are presented as mean ± SD (n = 4 independent replicates).

Table S1. Primers used for qRT-PCR. Related to Figure 1, Figure 4, and Figure 5.

Gene		Primer sequence (5’-3’)
Gapdh (Mouse)	Forward	CGTGCCGCCTGGAGAAACC
	Reverse	TGGAAGAGTGGGAGTTGCTGTTG
Alpl (Mouse)	Forward	CTTGGTGGTCACAGCAGTTGG
	Reverse	TTGACGTTCCGATCCTGAGTG
Runx2 (Mouse)	Forward	AAAACCAAGTAGCCAGGTTCAACG
	Reverse	TCAAGCTTCTGTCTGTGCCTTCTTG
Col1a1 (Mouse)	Forward	AGAAGATGTAGGAGTCGAGGGACC
	Reverse	TCATAGCCATAGGACATCTGGGAAG
Bglap (Mouse)	Forward	TCTTTCTGCTCACTCTGCTGACC
	Reverse	TGCTTGGACATGAAGGCTTTGTC
Cd206 (Mouse)	Forward	CACAGGATTAACCAGTTCCTTGAGC
	Reverse	GGTTTAGTGACACACAGCTCTTTCC
Arg1 (Mouse)	Forward	ACTGACTCTTCCATTCTTCTGGACC
	Reverse	AACAGAGTATGACGTGAGAGACCAC
Tgfb1 (Mouse)	Forward	TCTCTGTGGAGCTGAAGCAATAGTT
	Reverse	GGCACCATCCATGACATGAACC
Il10 (Mouse)	Forward	GAGTCCAGCAGACTCAATACACACT
	Reverse	TAAGCTCCAAGACCAAGGTGTCTAC
